# Antimycobacterial activity of intertidal sediment-derived bacteria from False Bay, South Africa

**DOI:** 10.3389/fmicb.2025.1745248

**Published:** 2026-01-15

**Authors:** Funanani Thagulisi, Lucinda Baatjies, Abhinav Sharma, Justice Trésor Ngom, Kudakwashe Nyambo, Tracey Jooste, Kudzanai Ian Tapfuma, Vuyo Mavumengwana

**Affiliations:** South African Medical Research Council Centre for Tuberculosis Research, Division of Molecular Biology and Human Genetics, Faculty of Medicine and Health Sciences, Stellenbosch University, Cape Town, South Africa

**Keywords:** antimycobacterial activity, intertidal marine sediments, intracellular antimycobacterial activity, marine bacteria, secondary metabolites, tuberculosis

## Abstract

Tuberculosis (TB), caused by *Mycobacterium tuberculosis*, remains a global health burden due to the pathogen’s ability to develop resistance to current treatment options. Consequently, drug discovery studies are essential for identifying new antimycobacterial agents with novel mechanisms of action. This study investigated the antimycobacterial activity of crude extracts derived from mixed culturable bacteria isolated from intertidal marine sediments. The bacterial diversity of the bioactive mixed cultures was characterized using 16S rRNA gene-based metagenomic analysis. Their pathogen-targeted effects were evaluated against *Mycobacterium smegmatis* mc^2^155 and *M. tuberculosis* H37Rv, and THP-1-derived macrophages infected with *M. smegmatis* mc^2^155. Of the 48 mixed bacterial crude extracts derived from 17 intertidal marine sediments, five-PPB1, GCR1, BB1, PPB2, and CR1-demonstrated strong antimycobacterial activity against *M. smegmatis* mc^2^155 and *M. tuberculosis* H37Rv with minimum inhibitory concentrations ranging from 31.25 to 62.50 μg/mL and 7.8125 to 15.625 μg/mL, respectively. At 62.50 μg/mL, CR1 significantly reduced the intracellular *M. smegmatis* mc^2^155 burden in THP-1-derived macrophages, resulting in 28.08 ± 4.25% mean decrease in bacterial survival (*p* < 0.0001) and 94.4% ± 1.14 mean growth inhibition. From the CR1 mixed cultures, nine axenic bacterial isolates were cultivated, and their resulting crude extracts were evaluated for bioactivity. The identified isolates included *Marinobacter maritimus*, *Psychrobacter celer*, *Pseudomonas benzenivor*, *Bacillus altitudinis*, *Bacillus aerius*, *Bacillus stratosphericus*, and *Paenibacillus glucanolyticus*. Metabolite profiling of axenic crude extracts identified several compounds, including tenacibactin B, maremycin D1, and tubercidine. These findings suggest that South African intertidal marine sediments host diverse microbial communities capable of producing novel antimycobacterial agents.

## 1 Introduction

Recent years have been referred to as the “resistance or post-antibiotic era” due to the growing prevalence of microbial strains that are resistant to most antimicrobial drugs (Ahmed et al., 2024). Tuberculosis (TB) treatment faces a similar challenge, as many TB strains now exhibit multi-drug resistance (MDR) to current antimycobacterial agents (Seung et al., 2015). In 2023, approximately 10.6 million people were diagnosed with TB, with more than half a million cases showing resistance to rifampicin, a cornerstone of first-line TB treatment (Ravikoti et al., 2025). This increasing prevalence has become a significant global health crisis that impacts not only health outcomes but also the economic and social landscapes of the affected countries (Salam et al., 2023). The situation is further worsened by the emergence of extensively drug-resistant (XDR) strains and resistance to newer treatments such as bedaquiline and delamanid (Shah et al., 2020), highlighting the urgent need for new antimycobacterial agents with novel mechanisms of action (Shah et al., 2020).

Across recent decades of research, bioprospecting of terrestrial microbial sources has led to the discovery of several key antibiotics, such as kanamycin, capreomycin, and streptomycin, all of which have significantly contributed to the treatment of TB (Cegielski et al., 2021; Freimane et al., 2023). However, the declining rate of antibiotic discoveries from terrestrial environments has prompted researchers to shift their focus toward marine ecosystems, which offer promising new opportunities for identifying novel therapeutic agents for TB (Bharathi and Lee, 2024; Srinivasan et al., 2021). Intertidal marine sediments, particularly within shallow coastal zones, are recognized as an underexplored reservoir of microbial diversity (Ishii et al., 2004; Verma et al., 2023). These environments provide a unique opportunity for the discovery of new microbial taxa that can produce a diverse array of chemically and biologically active metabolites (Ishii et al., 2004; Verma et al., 2023). The extreme environmental conditions prevalent in these habitats, such as fluctuating salinity, low temperatures, high tidal pressures, limited nutrient availability, and intermittent sunlight, create distinct ecological niches (Dalmaso et al., 2015; Fulke et al., 2025). These dynamic and often harsh conditions support a variety of microbial communities, including rare and uncultured species, as well as extremophilic microbes, which have the potential to produce novel bioactive compounds with significant therapeutic potential (Fulke et al., 2025).

Marine sediment-derived bacteria produce a wide variety of bioactive compounds that have shown promising antimycobacterial properties during preliminary screenings, including steroids, alkaloids, polyketides, terpenoids, and peptides (Jagannathan et al., 2021). As a result, marine sediments are valuable sources for isolating metabolically diverse bacteria that could yield new compounds effective against drug-resistant pathogens (Yao et al., 2023). For instance, abyssomicin C, a polyketide isolated from the bacterium *Verrucosispora maris* AB-18-032 found in the shallow marine sediments of the Japanese Sea, demonstrated notable antimycobacterial activity against *M. smegmatis* mc^2^155, *M. bovis* BCG, and *M. tuberculosis* H37Rv with minimum inhibitory concentration (MICs) of 2.6 μg/mL (Freundlich et al., 2010). Additionally, a series of macrolides, including niphimycin C and 17-O-methylniphimycin, produced by the bacterial strain *Streptomyces* sp. N-31, isolated from sediments in Heishijiao Bay, China, displayed potent activity against-resistant *M. tuberculosis* clinical strain FJ05-195, with MICs of 16 and 32 μg/mL, respectively (Wibowo et al., 2023).

South Africa boasts approximately 3,000 km of coastline shaped by the intricate interplay of the warm Agulhas and cold Benguela currents (Griffiths et al., 2010; Matobole et al., 2017). This dynamic marine environment supports a diverse range of ecosystems, ranging from subtropical coral reefs in the northeast to temperate kelp forests in the southwest (Griffiths et al., 2010; Matobole et al., 2017). Among these regions lies False Bay, a semi-enclosed embayment located on the southwestern coast, primarily influenced by the temperate Agulhas currents, while also receiving intermittent contributions from the nutrient-rich Benguela currents (Dufois and Rouault, 2012; Pfaff et al., 2019; Shannon and Chapman, 1983). The physicochemical differences generated by these currents result in False Bay exhibiting exceptionally high levels of biodiversity and endemism, particularly among intertidal microbial communities, making it a key area for bioprospecting new microbial taxa with therapeutic potential. Despite this abundant microbial diversity, the antimycobacterial potential of secondary metabolites derived from bacteria associated with the intertidal marine sediments of False Bay, South Africa remains largely uncharacterized, representing a significant yet untapped reservoir for the discovery of novel bioactive compounds. The present study evaluated the antimycobacterial and host-modulatory effects of methanolic crude extracts obtained from bacterial cultures isolated from marine sediments in the intertidal zones of False Bay, Cape Town, Western Cape, South Africa. *In vitro* antimycobacterial activity was evaluated against *M. smegmatis* mc^2^155 and *M. tuberculosis* H37Rv, as well as *ex vivo* in THP-1-derived macrophages infected with *M. smegmatis* mc^2^155.

## 2 Materials and methods

### 2.1 Collection of marine sediment samples

**Figure 1:**
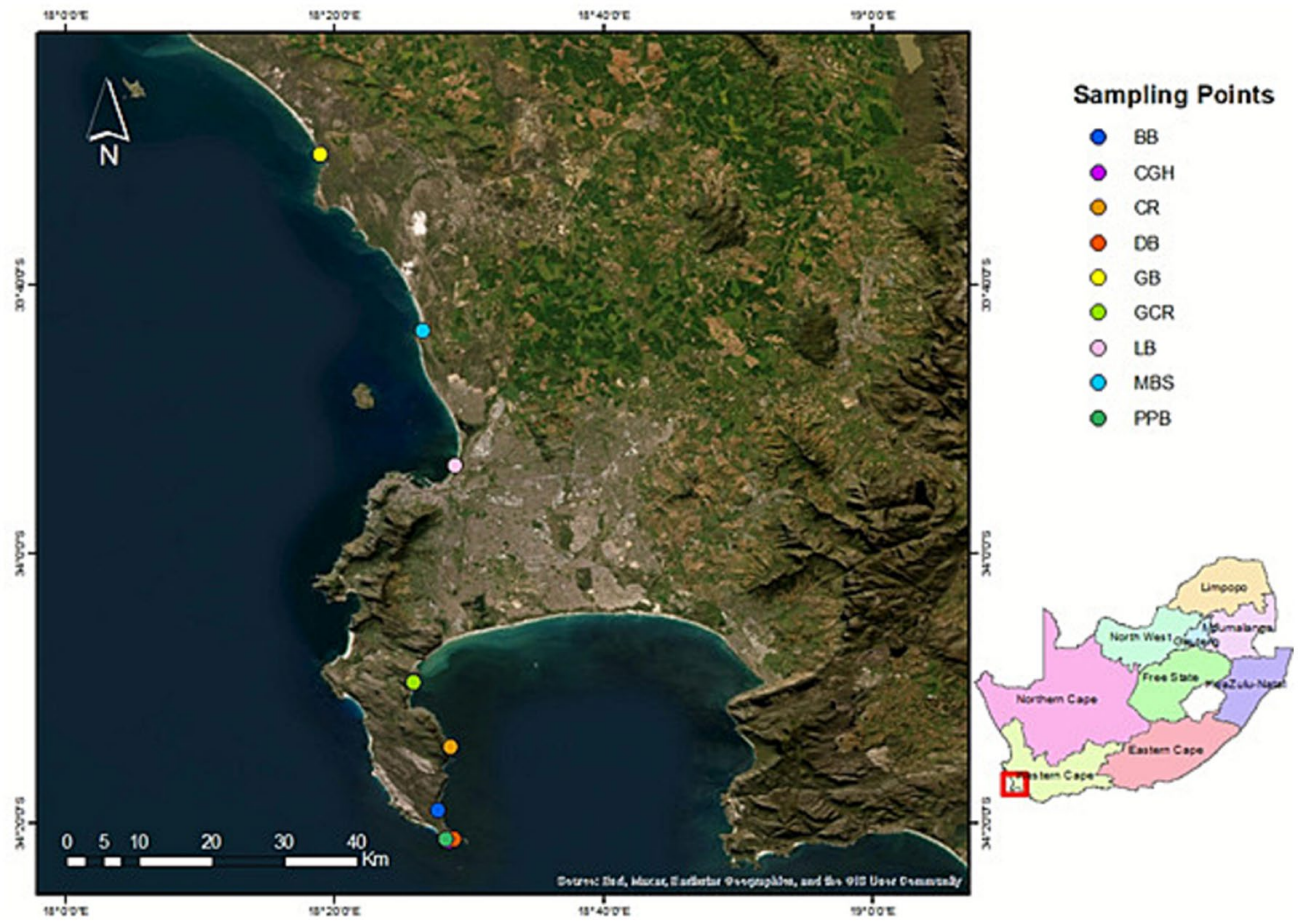
Sampling sites of the collected intertidal marine sediments. Created using ArcGIS Pro.

Collection of intertidal marine sediment samples, cultivation and isolation of bacterial strains, plating of bacterial suspensions, and extraction of bacterial secondary metabolites were conducted using the method described by Kashfi et al. (2020), with minor adjustments. A total of 17 marine sediment samples were collected along the intertidal shorelines of Buffles Bay, Castle Rock, Glencairn, Cape of Good Hope, Diaz, Grotto Bay, Melkbostrand, Lagoons, and Pappies Bank beaches in False Bay, Cape Town, South Africa, during the late winter of 11 August 2023 (Figure 1). During this time of the year, the surface seawater temperature in False Bay is at its coldest, ranging between 10 °C and 13 °C. Sediment samples were collected in duplicate, approximately 6.7 m from the shoreline using sterile 50 mL centrifuge tubes to prevent cross-contamination between sampling sites. After collection from all sites, the samples were stored in a plastic zip-lock bag with ice and transported to the laboratory. Upon arrival at the laboratory, bacteria were isolated from the sediment samples, and the remaining portions were stored at 4 °C. The geographic coordinates for each sampling site are listed in Supplementary Table S1.

### 2.2 Isolation of bacteria from intertidal marine sediment samples

To isolate bacteria from the collected sediment samples, about 10 g of each sediment sample was prepared for processing. To prevent the bacteria from rupturing, 10 mL of 1X phosphate-buffered saline (PBS, Gibco) was added to each sample. Sediment samples were then dislodged by sonication using a Virsonic 475 ultrasonic cell disrupter system (DC150H) with a 20 KHz sonic probe at 109 μm amplitude for 20 min, followed by centrifugation (Centurion) at 1000 rpm for 10 min. The resulting supernatants were decanted into new sterile 50 mL centrifuge tubes using sterile serological pipettes (NEST).

### 2.3 Cultivation and enrichment of mixed bacterial cultures

Bacterial cultivation was performed in triplicate for each sediment sample on solid agar media, which included Luria-Bertani agar (LBA, Sigma), tryptic soy agar (TSA, Sigma), and Actinomycetes (Sigma). The agar media were prepared using 1 L of distilled water. The cultivated bacterial isolates were then plated directly onto their respective agar plates using a sterile loop. The inoculated agar plates were incubated at 37 °C in an UN-1-TROL co-incubator 329 for approximately 7 days, with continuous monitoring of colony growth. After incubation, mixed bacterial colonies were inoculated into LB, tryptic soy, and actinomycetes broths and then cultured at 37 °C on a rotary shaker for an additional 7 days to promote the production of secondary metabolites. This was achieved by measuring the optical density (O.D.) of the bacterial cultures every 24 hours (h) throughout the incubation period.

### 2.4 Extraction of secondary metabolites from bacterial cultures

To extract secondary metabolites, 15 g of XAD4 Amberlite resin (Supelco) was added to 500 mL of unfiltered bacterial broth cultures, allowing absorption of both extracellular and cell-associated metabolite. Prior to use, the XAD4 Amberlite resin was washed twice with autoclaved distilled water and air-dried at room temperature for 48 h. Resin-treated cultures were subsequently incubated at 37 °C with agitation on a rotary shaker incubator for 24 h. After incubation, the resin was filtered out from the bacterial cultures using Whatman Grade 1 qualitative filter paper (Sigma). Bacterial secondary metabolites were eluted from the resin using three different solvents of varying polarity: hexane (Alpha Chemica), ethyl acetate (Alpha Chemica), and methanol (Alpha Chemica). The extracted metabolites were then allowed to dry at room temperature in a fume hood for approximately 14 days to yield concentrated crude extracts. These extracts were subsequently used for all biological assays.

### 2.5 Antimycobacterial activity screening of bacterial crude extracts

The antimycobacterial activity of the bacterial crude extracts was assessed using a method previously described by Tapfuma et al. (2023), with minor adjustments. Methanolic, hexanoic, and acetonic bacterial crude extracts were initially dissolved in 100% dimethyl sulfoxide (DMSO) and subsequently diluted to 1% DMSO. Bacterial crude extracts with sufficient yield were selected for screening against *Mycobacterium* strains, which were maintained in Middlebrook 7H9 broth medium containing 0.05% tween 80, 0.5% glycerol, and 10% oleic acid-albumin-catalase (OADC) supplements. Initial screening was conducted against *M. smegmatis* mc^2^155 and *M. tuberculosis* H37Rv at a concentration of 1 mg/mL in 96-well microplates as outlined by Tapfuma et al. (2023). Treatments were incubated for three and 6 days for *M. smegmatis* mc^2^155 and *M. tuberculosis* H37Rv, respectively.

The antimycobacterial activity of the bacterial crude extracts was determined by adding 30 μL of 0.02% resazurin dye (Sigma) to each well, followed by a further incubation at 37 °C for 4 h for *M. smegmatis* mc^2^155, and 24 h for *M. tuberculosis* H37Rv. After incubation, 96-well microplates (NEST), were qualitatively assessed for antimycobacterial activity, which was noted by the reduction of resazurin from blue to pink (resorufin) for extracts with no bioactivity. Extracts that exhibited antimycobacterial activity were further evaluated using a resazurin-based broth-microdilution assay to determine their minimum inhibitory concentrations (MICs) range. This assay was also carried out using 96-well microplates (NEST), following the same previously outline protocol. Individual MICs were then determined, reporting the concentration range (highest to lowest) at which cellular growth was inhibited. Isoniazid (INH), a first-line anti-tubercular agent, was used as a positive control for all antimycobacterial assays.

### 2.6 Metagenomic profiling of bacterial communities associated with mixed bioactive crude extracts

To characterize and identify the taxonomic composition, diversity, and relative abundance of the bacterial communities associated with mixed cultures of the bioactive crude extracts, metagenomic sequencing was employed. DNA extraction and sequencing were conducted at Inqaba Biotechnological Industries in Pretoria, South Africa.^1^ A full detailed protocol for sample preparation, DNA extraction, and 16S ribosomal RNA (rRNA) sequencing of the bioactive mixed bacterial cultures is provided in the supplementary methods. The raw 16S rRNA gene sequences from mixed cultures were assessed for quality using FASTQC (version 0.11.8). Taxonomic classification and relative abundance analyses were subsequently performed following the methodology previously described by Lu et al. (2022), with minor adjustments. Taxonomic classification of the raw 16S rRNA gene sequences was carried out using Kraken2 (version.2.1; https://github.com/DerrickWood/kraken2/), comparing k-mer content against entries in the bacterial genome database. Species abundance estimates, indicating the number of reads per species, were derived from the Kraken2 classification reports using Bracken (version 2.6.2; https://github.com/jenniferlu717/Bracken), employing Bayesian re-estimation to improve accuracy. The output files from Bracken were then used for visualization with KrakenTools (version 1.1) and Pavian (version 1.0). The latter facilitated the creation of Sankey visualizations to effectively illustrate comparisons in sample composition and read counts across different samples.

### 2.7 Assessment of the intracellular antimycobacterial activity of bioactive bacterial crude extracts in THP-1-derived macrophages

Bioactive crude extracts that exhibited antimycobacterial activity were further evaluated for their ability to reduce the intracellular *M. smegmatis* mc^2^155 burden in THP-1-derived macrophage. Prior to this assessment, the extracts were screened for cytotoxicity against THP-1-derived macrophages using a 3-(4,5-dimethylthiazol-2-yl)-2,5-diphenyltetrazolium bromide (MTT) assay to determine their non-cytotoxic concentration for treating infected THP-1-derived macrophages.

#### 2.7.1 Growth, maintenance, and differentiation of THP-1 cells

A cryopreserved stock of THP-1 cells (1 mL) was thawed at 37 °C for 3 min and thereafter transferred to 5 mL of pre-warmed complete RPMI 1640 medium with L-Glutamine (Sigma), supplemented with 10% fetal bovine serum (FBS) (Gibco, Thermo-scientific). To remove any residual DMSO (Sigma), THP-1cells were centrifuged at 1000 rpm for 5 min, resuspended in 5 mL complete RPMI medium, and incubated in a 25 cm^2^ flask at 37 °C with 5% CO_2_ for 4 days. THP-1 cell cultures were visually examined for cell growth and bacterial contamination under an inverted phase contrast microscope (Zeiss, Nikon) throughout the incubation period. Once confluency was reached, THP-1 cell suspensions were subsequently transferred to a 15 mL falcon tube, centrifuged at 1000 rpm for 5 min, and the resulting pellet was resuspended in 1 mL of complete RPMI medium and sub-cultured in a 75 cm^2^ tissue culture flask (Greiner, Lasec) containing 20 mL of complete RPMI medium. Subsequently, sub-cultured THP-1 cells were counted using a Neubauer hemocytometer by diluting the suspension 5-fold, mixing 20 μL of the resuspended pellet with 80 μL of trypan blue (Sigma). The cell culture was then diluted to a density of 2 × 10^5^ cells/mL for THP-1 infection assays and 1 × 10^4^ cells per well in 200 μL aliquots for cytotoxicity screening and ApoTox Glo Triplex assay (Promega). Additionally, 50 ng/mL phorbol 12-myristate acetate (PMA, Sigma) was added to differentiate the cells. THP-1 cells were then seeded and subsequently incubated at 37 °C in a humidified incubator (ESCO Vivid Air) with 5% CO_2_ for 3 days to allow for cell differentiation.

#### 2.7.2 Cytotoxicity screening of bioactive bacterial crude extracts against THP-1-derived macrophage cells

THP-1-derived macrophages were maintained in complete RPMI medium supplemented with 10% FBS, and seeded at 10,000 cells/well in 200 μL aliquots in a 96 well plate and allowed to attach overnight in a humidified incubator (ESCO Vivid Air) with 5% CO_2_ at 37 °C. Bioactive bacterial crude extracts were prepared in complete RPMI medium and subsequently added to the wells at 31.25 and 62.5 μg/mL, while INH was screened at 15.63 μg/mL. Following treatment with the bioactive bacterial crude extracts, THP-1-derived macrophages were incubated for 48 h, before removing the spent-medium and adding 200 μL of 0.5 mg/mL MTT to each well, followed by a further 4 h incubation. After incubation, the MTT was removed and 100 μL of DMSO was added to the THP-1-derived macrophages and the absorbance were measured in a microtiter plate reader (FLUOstar Omega, BMG Labtech,) at 540 nm. The plates were agitated for 1 min before the absorbance (Abs) was read. The percentage of cell viability was calculated using the following formula:

$$\%\text{Of cell viability}=\frac{\left(\mathrm{Abs}\;\text{of treated cells}-\mathrm{Abs}\;\text{of blank}\right)}{\left(\mathrm{Abs}\;\text{of untreated cells}-\mathrm{Abs}\;\text{of blank}\right)}\times 100\%$$

#### 2.7.3 Infection of THP-1-derived macrophage cells with *M. smegmatis* mc^2^155 and subsequent treatment with bacterial crude extracts

On the day before the infection of THP-1-derived macrophages, 1 mL of *M. smegmatis* mc^2^155 was inoculated in a 10 mL Middlebrook 7H9 broth supplemented with 0.05% Tween 80, 0.5% glycerol and 10% of OADC, and cultured overnight to reach an OD_600nm_ of 1–1.5 (whereby OD_600nm_ 1.0 = 1 × 10^8^). On the day of the infection, 10 mL of the bacterial culture was centrifuged at 4,000 × *g* for 10 min. The bacterial culture was washed twice with 10 mL of pre-warmed PBS, and the pellet was subsequently re-suspended in 5 mL of pre-warmed complete RPMI medium. To disrupt any cell clumps, the resuspended bacterial culture was sonicated using a Virsonic 475 ultrasonic cell disrupter system (DC150H) for 12 min at 37 °C. The sonicated bacterial culture was then filtered through a 40 μm filter (falcon) to remove any clumps in preparation for intracellular infection assessment. THP-1 cells were maintained in complete RPMI medium supplemented with 10% FBS and then seeded at a density of 2 × 10^5^ cells per well in a 24 well plate. After seeding, THP-1 cells were differentiated with 50 ng/mL of PMA for 72 h, after which spent medium was removed and the THP-1-derived macrophages were washed twice with 1 mL of pre-warmed 1X PBS. Fresh complete RPMI medium (1 mL) was then added to each well of the 24-well plates and subsequently incubated (ESCO Vivid Air with 5% CO_2_) at 37 °C overnight. After incubation, the spent medium was removed, and a culture of *M. smegmatis* mc^2^155 was diluted in 5 mL of complete RPMI medium to achieve a multiplicity of infection (MOI) of 2:1. One milliliter of the diluted bacterial culture was then added to the THP-1-derived macrophages in triplicate to initiate infection. Infected THP-1-derived macrophages were then incubated at 37 °C in a humidified incubator with 5% CO_2_ for 3 h to allow for bacterial-cell interaction. After incubation, the extracellular bacterial suspension was removed, and the infected THP-1-derived macrophages were treated with 1 mL of penicillin–streptomycin (10,000 U/mL) and subsequently incubated for 1 h to remove any extracellular bacteria. After incubation, the infected THP-1-derived macrophages were washed twice with 1 mL of 1 X PBS and treated with 0.63 μL of each bioactive bacterial crude extract in triplicate and further incubated for 24 h. The effects of each bacterial crude extract on the intracellular *M. smegmatis* mc^2^155 burden in THP-1-derived macrophages were assessed at 6 h intervals over a 24 h period.

#### 2.7.4 Count of viable colonies

Infected THP-1-derived macrophages were treated with bioactive bacterial crude extracts in triplicates. For each extract, colony forming units (CFUs) were counted every 6 h over a 24 h treatment period, following an initial incubation to allow colony formation. At each 6 h time point, infected THP-1-derived macrophages were treated with bioactive bacterial crude extracts. INH, a first-line pro-drug for TB, was used as positive control. Following antibiotic treatment, infected THP-1-derived macrophages were washed three times with 5 mL of 1 X PBS and lysed by adding 500 μL of sterile water per well, followed by a serial dilution of 10^−1^ to 10^−4^ in PBS supplemented with 0.05% Tween-80. Aliquots of 50 μL from each dilution were plated in quadruples on Middlebrook 7H10 medium supplemented with 5% glycerol and 10% of OADC. Plates were incubated at 37 °C, and colonies were counted after 2–4 weeks. Colonies counted in 10^−3^ were eventually used for calculations, results were reported as CFU/mL.

#### 2.7.5 Determination of cell death mechanism using Annexin V-FITC and PI-stained cells

After 24 h incubation of the infected and extract-treated THP-1-derived macrophages, 50 μL of Accutase was added to each well for 10 min or until cell detachment. To facilitate recovery of the infected-treated THP-1-derived macrophages, 1 mL of complete RPMI medium was added to each well, followed by incubation at 37 °C in a humidified incubator (ESCO Vivid Air) with 5% CO_2_ for 1 h. Infected-treated THP-1-derived macrophages were subsequently transferred to polypropylene flow cytometry tubes and collected by centrifugation at 1500 rpm for 5 min at 4 °C. Cell pellets were then washed with ice-cold complete RPMI medium and centrifuged again at 1500 rpm for 5 min at 4 °C. After centrifugation, the cells were stained using Annexin V-FITC/propidium iodide (PI) apoptosis detection kit (Invitrogen, ThermoFisher Scientific). Subsequently, the cells were resuspended in an ice-cold 1x binding buffer. To each tube, 1 μL of Annexin V and 5 μL of PI were added. Control tubes containing single stains of PI, Annexin V, and their combination were prepared. The mixtures were then incubated in the dark for 15 min. Following incubation, 400 μL of 1x Annexin-binding buffer was added to each well and mixed gently. Samples were then analyzed using a BC DxFlex flow cytometer (Beckman Coulter, USA).

### 2.8 Isolation and identification of axenic cultures from mixed bacterial populations

Axenic bacterial isolates were cultivated from mixed cultures of the bioactive bacterial crude extracts that demonstrated minimal cytotoxicity profile and significantly reduced the intracellular *M. smegmatis* mc^2^155 burden in THP-1-derived macrophages. This was achieved by subculturing the mixed culture onto agar plates at varying dilutions (10^−1^ −10^−4^) until an axenic culture was obtained. The obtained axenic isolates were subsequently sent to Inqaba Biotechnological Industries (Pretoria, South Africa; https://inqababiotec.co.za/) for 16S rRNA sequencing and bacterial identification. Briefly, genomic DNA was extracted from cultures of pure bacterial isolates using the Quick-DNA™ Fungal/Bacterial Miniprep Kit (Zymo Research, Catalogue No. D6005). The 16S target region was amplified by Polymerase Chain Reaction (PCR) using two universal primers, 27F (5′-AGA GTT TGA TCM TGG CTC AG-3′) and 1492R (5′-CGG TTA CCT TGT TAC GAC TT-3′). PCR reactions were conducted in 0.2 mL microcentrifuge tubes. Each reaction had a total volume of 50 μL, consisting of 25 μL of One Taq (2X) Master mix, 1.0 μL of reverse and forward primers, 1.0 μL of Genomic DNA, and 22 μL of nuclease-free water. The reaction mixtures were placed in a thermocycler with an initial denaturation step at 94 °C for 5 min, followed by 35 cycles at 94 °C for 30 s, annealing at 50 °C for 30 s, and extension at 68 °C for 1 min, with a final extension at 68 °C for 10 min. The integrity of the PCR products was evaluated by agarose gel electrophoresis in a 1% agarose gel (CSL-AG500, Cleaver Scientific Ltd) stained Bluelight DNA dye. The NEB fast ladder was used on all gels (N3238) as size standard. The PCR fragments were enzymatically purified using the ExoSAP procedure (NEB M0293L; NEB M0371). PCR amplicons were purified using the ZR-96 DNA Sequencing Clean-up Kit™ (Zymo Research, Cat. No. D4050) and sequenced in both forward and reverse directions using the BrilliantDye™ Terminator Cycle Sequencing Kit v3.1 (Nimagen, Cat. No. BRD3-100/1000). Sequencing was perfomed using an ABI 3730xl Genetic Analyzer (Applied Biosystems, Thermo Fisher Scientific). The raw chromatogram files (.abi) were visualized using FinchTV (v.14; https://finchtv.software.informer.com/1.4/). Forward and reverse reads were then assembled into consensus sequences using CLC Bio Main Workbench from Qiagen. The resulting consensus sequences were analyzed using BLASTn on the NCBI BLAST platform.^2^ Matches were considered significant if they exhibited ≥99% query coverage and ≥99% sequence identity. Procedures for bacterial isolation, cultivation, maintenance, extraction of bacterial secondary metabolites, and subsequent cytotoxic and intracellular antimycobacterial screening of the axenic bacterial crude extracts were conducted following the established protocols previously applied to mixed bacterial crude extracts.

### 2.9 Untargeted metabolite profiling of axenic crude extracts using LC-QTOF-MS

Metabolic profiling of axenic bacterial extracts was performed using a liquid chromatography coupled with quadrupole time-of-flight mass spectrometry (LC-QTOF-MS/MS) in positive mode. Each crude bacterial sample (1 mg) was dissolved in 1 mL of HPLC grade methanol and sonicated for 10 min. The samples were then filtered through a 0.22 μm polyvinylidene fluoride (PVDF) membrane syringe filters into a 1 mL LC autosampler vials. High-quality mass spectra data of the crude extracts were acquired using an AB Sciex® X500R QTOF coupled with an AB Sciex® Exion LC system. Metabolite separation was achieved on a Kinetex® C18 column (5 μm particle size, 100Å pore size, 150 mm × 6 mm dimensions). The mass spectrometer was operated using a delustering potential of 80 V, curtain gas (N_2_) at 25 psi, ion spray voltage at 5,500 V, and source temperature at 450 °C. Ion source gases 1 and 2 were maintained at 45 and 55 psi, respectively. Collision energy was set to 10 electron volts (eV) for MS scans and varied from 20 to 50 Ev for MS/MS scans. The threshold for the independent data acquisition (IDA) was set at 50 cycles per second. The aqueous mobile phase was 1 mM ammonium formate, and the organic phase was 0.5% formic acid in methanol. The gradient elution for the organic mobile phase started at 2% and increased to 98% over 25 min, held for 5 min, then returned to 2% over 5 min before re-equilibration. The flow rate was set at 700 μL/min, and the total run time was 35 min. Spectral data were acquired in positive centroid mode within 50 to 1,500 Da mass range. Electrospray ionisation was performed with a cone voltage of 15 V and a capillary voltage of 2.5 Kv. Nitrogen desolvation was set 650 L/h, with a desolvation temperature of 275 °C.

The raw data containing spectral information were converted to abf format using the ABF converter (version.1.2, http://www.reify.cs.com/AbfConverter). The converted files were processed using MS-Dial (version.4.24, http://prime.psc.riken.jp/compms/msdial/main.html), with the following parameters settings: MS1 range, m/z 50–1,250; MS1 and MS2 tolerances, 0.01 and 0.025 Da, respectively; [M + H] ^+^ adducts ions with a mass error of <7.0. Tentative metabolite of molecular formula and structure elucidation of the retrieved bacterial metabolites was done using MS-FINDER,^3^ with both MS1 and MS2 tolerance parameters set at 0.01 Da; isotopic ratio tolerance of 20% and an in-silico fragmentation tree depth of 2. Metabolite identities were annotated based on database matching with KNapSack, ChEBI (Biomolecules), PubChem, and UNDP (natural products).

### 2.10 Statistical analysis

Absorbance values from the MTT assay and CFU counts from the THP-1-derived macrophage infection assays were analyzed using Microsoft Excel 2023 and GraphPad Prism 8. A two-way repeated measures ANOVA were performed to assess the effects of bacterial crude extracts, time, and their interaction, with a significance threshold set at a *p*-value of less than or equal to 0.05.

## 3 Results

### 3.1 Isolation and bioactivity assessment of mixed bacterial crude extracts

Of the 17 intertidal marine sediment samples collected, 48 mixed bacterial crude extracts with sufficient biomass were isolated from 153 agar plates. Among these, nine extracts (BB1, PPB1, CR1, GCR1, PPB2, GB2, LB1, MBS1, and DB2) exhibited significant inhibitory effects against the tested *Mycobacterium* strains. Bioactive bacterial crude extracts were prepared using methanol as the solvent of extraction. In contrast, crude extracts prepared with hexane and ethyl acetate did not exhibit antimycobacterial activity, likely due to the limited solubility of polar metabolites in non-polar solvents. Of the nine bioactive bacterial crude extracts, five (BB1, PPB1, CR1, GCR1, and PPB2) displayed strong inhibitory effects against both *M. smegmatis* mc^2^155 and *M. tuberculosis* H37Rv, with MIC ranges of between 31.25 and 62.50 μg/mL for *M. smegmatis* mc^2^155 and 7.812 to 15.625 μg/mL for *M. tuberculosis* H37Rv. In comparison, crude extracts GB2, LB1, and DB2 displayed weaker antimycobacterial activity, with MICs ranging from 62.50 and 125 μg/mL against *M. smegmatis* mc^2^155 and 31.25 and 62.50 μg/mL against *M. tuberculosis* H37Rv. Isoniazid (INH), used as positive control, demonstrated the strongest bioactivity against *M. smegmatis* mc^2^155, with MIC ranges of between 15.625 and 31.25 μg/mL, and demonstrated comparable MIC ranges to the mixed bioactive bacterial crude extracts against *M. tuberculosis* H37Rv (Table 1). The susceptibility threshold for *M. smegmatis* mc^2^155 was set at an MIC range of between 31.25 and 62.50 μg/mL, while a threshold of greater than 31.25 μg/mL was established for *M. tuberculosis* H37Rv. Extracts that met these MIC susceptibility thresholds for both *M. smegmatis* mc^2^155 and *M. tuberculosis* H37Rv were selected for subsequent 16S rRNA gene-based metagenomic analysis, and intracellular infection screening assays.

**Table 1:** Minimum inhibitory concentrations (MICs) of the bioactive bacterial crude extracts against *M. smegmatis* mc^2^155 and *M. tuberculosis* H37Rv strain.

Sample code	Culture type	Extraction solvent	Agar type	Antimycobacterial activity	
				Test organism MIC (μg/mL)	
				Mc^2^155	H37Rv
PPB1	Mixed	Methanol	LB	31.25–62.50	7.812–15.625
BB1	Mixed	Methanol	TSA	31.25–62.50	7.812–15.625
CR1	Mixed	Methanol	LB	31.25–62.50	7.812–15.625
PBB2	Mixed	Methanol	Actinomycetes	31.25–62.50	7.812–15.625
GCR1	Mixed	Methanol	LB	31.25–62.50	7.812–15.625
LB1	Mixed	Methanol	LB	>62.50	>15.625
GB2	Mixed	Methanol	LB	>62.50	>15.625
DB2	Mixed	Methanol	LB	>62.50	>15.625
CR1-1	Axenic	Methanol	LB	7.815–15.625	3.907–7.815
CR1-2	Axenic	Methanol	LB	7.815–15.625	3.907–7.815
CR1-3	Axenic	Methanol	LB	7.815–15.625	3.907–7.815
CR1-4	Axenic	Methanol	LB	7.815–15.625	3.907–7.815
CR1-5	Axenic	Methanol	LB	7.815–15.625	3.907–7.815
CR1-6	Axenic	Methanol	LB	62.50–125	31.125–62.50
CR1-7	Axenic	Methanol	LB	62.50–125	31.125–62.50
CR1-8	Axenic	Methanol	LB	62.50–125	31.125–62.50
CR1-9	Axenic	Methanol	LB	62.50–125	31.125–62.50
INH	—	—	—	16.25–31.25	7.812–15.63

Isoniazid (INH) was used as reference first-line TB drug/positive control.

### 3.2 Metagenomic characterization and community composition of bioactive mixed bacterial samples

**Figure 2:**
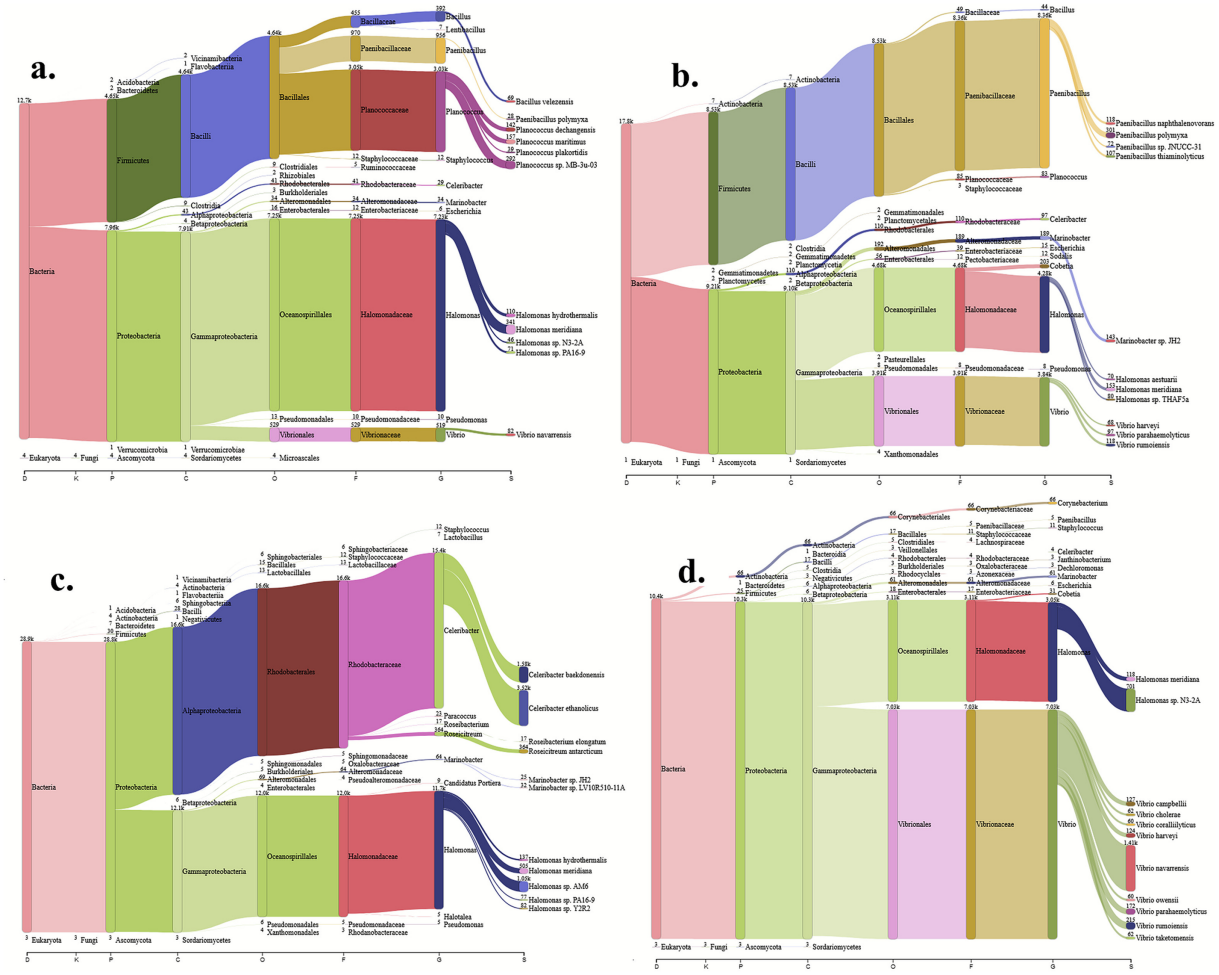
Sankey diagram produced by the Pavian with the total number of corrected reads as estimated with Bracken for all bioactive mixed bacterial samples, BB1 **(a)**, GCR1 **(b)**, CR1 **(c)**, and PPB2 **(d)**, combined at phylum, class, order, family, genus, and species level.

Mixed bacterial culture samples BB1, PPB1, CR1, GCR1, and PPB2 were selected for metagenomic analysis using 16S rRNA gene sequencing to determine and characterize their taxonomic composition, species diversity, and abundance. However, since PPB1 and PPB2 were collected from the same environmental niche, only PPB2 was included. The microbial communities identified in each sample are summarized (Figure 2). Analysis of the 16S rRNA gene sequences showed that all the detected DNA represented bacterial phyla, indicating that the samples comprised exclusively of only bacterial communities. Proteobacteria represented the most abundant phylum across all samples, accounting for 63% in BB1, 51% in GCR1, 100% in CR1, and 99% in PPB2. In addition to Proteobacteria, GCR1 and BB1 showed evidence of the Firmicutes phyla, comprising 49 and 37% of their respective communities. While Proteobacteria emerged as the dominant phylum, further analysis at a finer taxonomic resolution revealed that Gammaproteobacteria was the most dominant class across all the mixed culture samples, constituting 54% of BB1, 46% of GCR1, 72% of CR1, and 99% of PBB2. The class Bacilli followed, representing 43% of BB1, 54% of GCR1, 2.04% of CR1, and 0.09% of PPB2 population.

The Halomonadaceae family proved to be the most dominant across all samples, making up 59% of the BB1 population, and 25%, 42%, and 30% of GCR1, CR1, and PPB2, respectively. In contrast, GCR1 showed predominance of Paenibacillaceae family-related bacteria, which accounted for about 49% of its bacterial population, while 26% of its remaining bacterium belonged to the family Vibrionaceae. CR1 was characterized by a high prevalence Rhodobacteraceae, accounting for 58% of its population. In PPB2, Vibrionaceae dominated, constituting 69% of its bacterial population. At the genus level, *Halomonas*, *Paenibacillus*, and *Vibrio* were the most prevalent taxa across all samples. *Halomonas* represented 47% of the BB1 population, 46% of GCR1, and 17% of PPB2. *Paenibacillus* accounted for 53% of GCR1, while *Vibrio* comprised 75% of PPB2 and 17% of GCR1. However, a substantial proportion of genera remains unclassified, with BB1, CR1, and PPB2 comprising 31%, 35%, and 6% of the unknown genera, respectively.

**Figure 3:**
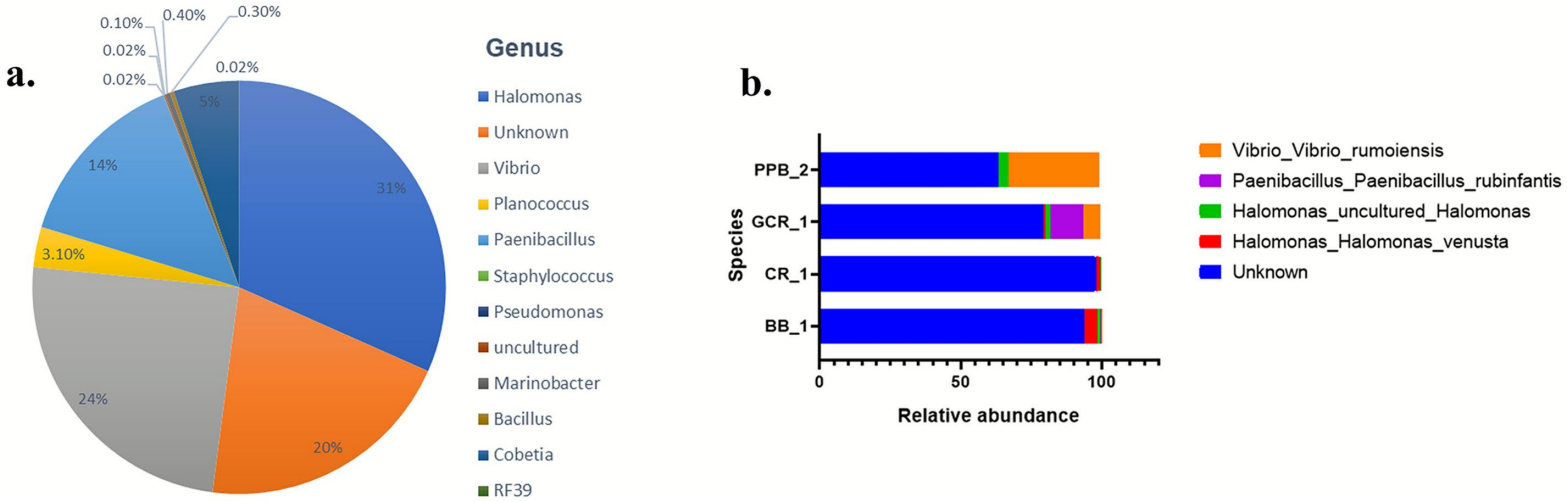
**(a)** Overall relative abundance of bacterial genera across mixed bacterial samples, and **(b)** relative abundance of species within individual mixed bacterial samples.

Overall, the genus *Halomonas* emerged as the predominant microbial group, constituting 31% of the total identified bacterial populations across all samples. *Vibrio* was the second most prevalent genus, representing 24% of the bacterial composition. Notably, approximately 20% of the bacterial population remained classified as unidentified genera. Lesser represented genera included *Paenibacillus*, *Cobetia*, and *Planococcus*, which accounted for 14%, 5%, and 3% of the total populations, respectively, as shown in Figure 3a. Analysis of species diversity within individual mixed cultures revealed that GCR1 exhibited a higher level of diversity, being dominated by four distinct species. Conversely, the cultures of CR1, BB1, and PPB2 displayed reduced diversity, each characterized by a limited number of dominant species. A remarkable finding across all samples was the substantial proportion of unclassified species, indicating the presence of novel or less-characterized microbial taxa (Figure 3b).

### 3.3 Inhibition of intracellular *M. smegmatis* mc^2^155 burden in THP-1-derived macrophages by bioactive bacterial crude extracts

**Figure 4:**
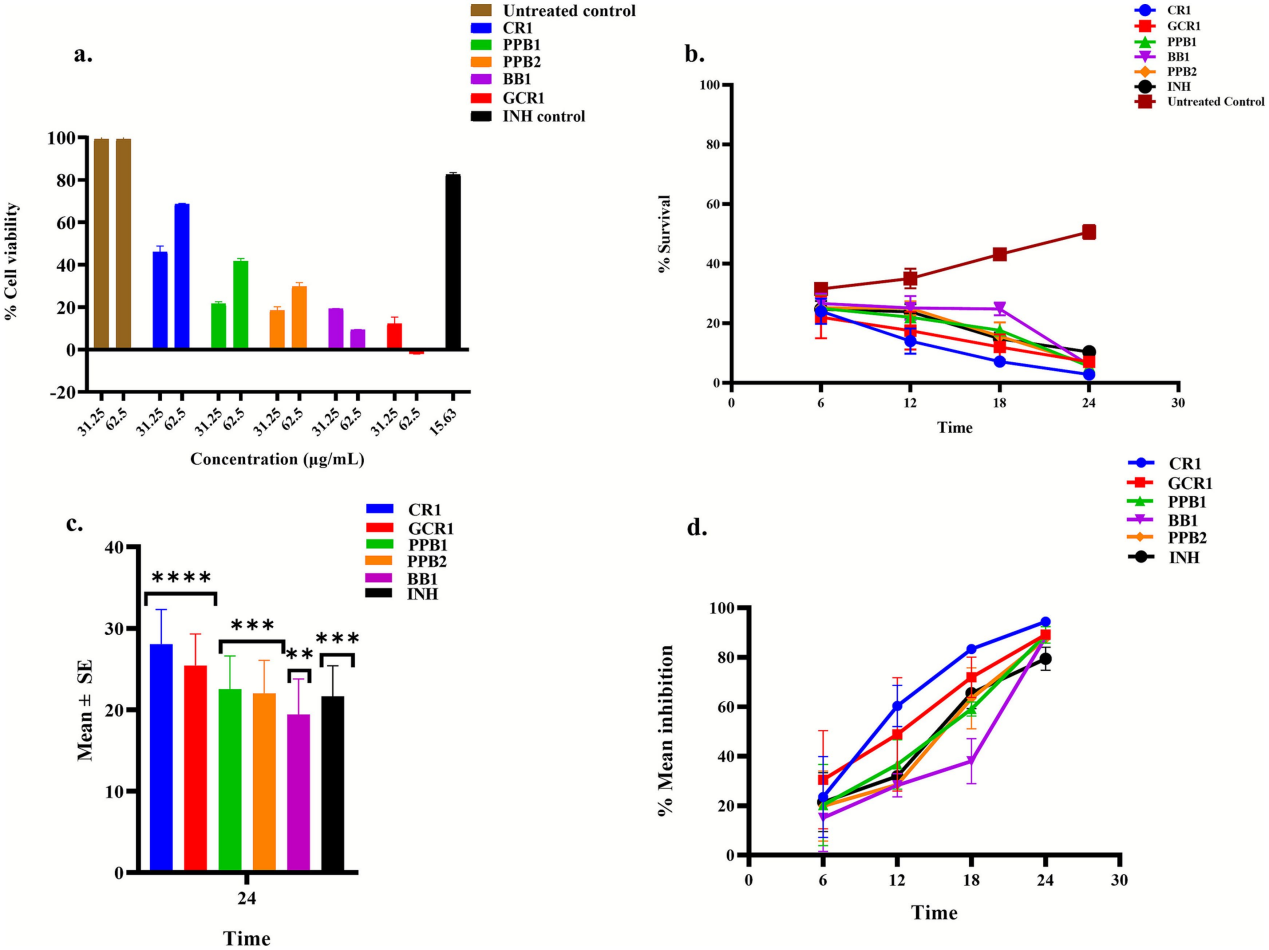
Effect of treatment with mixed bioactive bacterial crude extracts on uninfected and infected THP-1-derived macrophage cells. **(a)** Cytotoxicity (% cell viability) of uninfected THP-1-derived macrophages post treatment with mixed bacterial crude extracts. Screening was conducted at 31.25 and 62.50 μg/mL, with results represented as mean ± SD from triplicate wells (*n* = 2). **(b)** Percentage survival of intracellular *M. smegmatis* mc^2^155 burden in THP-1-derived macrophages post-treatment with mixed bacterial crude extracts. The results are shown as mean ± SD from triplicate wells (*n* = 2). **(c)** Mean reduction of the intracellular *M. smegmatis* mc^2^155 burden in THP-1-derived macrophages post-treatment with mixed bacterial crude extracts. Bars represent SE. Statistical significance was determined using Dunnett’s post-hoc multiple tests of comparisons (**p < 001, ***p < 0.001, ****p < 0.0001). **(d)** Time-dependent percentage mean inhibition of intracellular *M. smegmatis* mc^2^155 burden in THP-1-derived macrophages post-treatment with mixed bacterial crude extracts. Data represents mean ± SD of triplicate wells (*n* = 2); error bars indicate replicate variability. Treatments of were conducted for 24 h, with Isoniazid (INH) used as a positive control in all experiments.

Prior to evaluating the efficacy of the extracts in reducing the intracellular *M. smegmatis* mc^2^155 burden in THP-1-derived macrophages, a cytotoxicity assay was conducted to determine the non-toxic concentrations for the host cells. This assay was carried out at concentrations of 31.25 and 62.50 μg/mL, using 0.25% DMSO as the untreated control. INH was used as a control for assessing host cell tolerance. Among the screened bioactive bacterial crude extracts, BB1, PPB1, CR1, GCR1, and PPB2, CR1 exhibited a minimal cytotoxicity profile, maintaining 68.86% cell viability at 62.50 μg/mL. In contrast, PPB1 and PPB2 showed marked cytotoxicity, with cell viabilities of 41.63 and 29.78%, respectively. BB1 and GCR1 were deemed highly toxic, yielding cell viability of 9.32% and −1.97%, respectively. In comparison, treatment with INH exhibited minimal cytotoxicity, maintaining cell viabilities of greater than 82% at both concentrations tested (Figure 4a). The cytotoxicity profiles observed in this study demonstrated a concentration-dependent effect, with lower toxicity observed at 62.50 μg/mL compared to 31.25 μg/mL for most extracts. Despite differences in cytotoxicity profiles, all five bioactive bacterial crude extracts were selected for subsequent evaluation of their ability to reduce the intracellular *M. smegmatis* mc^2^155 burden in THP-1-derived macrophages.

THP-1-derived macrophages infected with *M. smegmatis* mc^2^155 were treated with 0.63 μL of each bioactive bacterial crude extracts for 24 h, and their efficacy in reducing the intracellular burden was assessed by using a two-way repeated measures ANOVA, with treatment and time as factors. The analysis revealed significant main effects of treatment on the intracellular *M. smegmatis* mc^2^155 burden (*p* = 0.0001) and time (*p* < 0.0001), as well as significant treatment x time interaction (*p* < 0.0001). A post-hoc Dunnett’s test demonstrated that GCR1, PPB1, BB1, PPB2, CR1, and INH significantly reduced the intracellular *M. smegmatis* mc^2^155 burden in THP-1-derived macrophages post 24 h treatment (Figure 4b). Among the screened extracts, CR1 exhibited the lowest percentage survival of *M. smegmatis* mc^2^155 in THP-1 derived macrophages with a mean reduction of 28.08 ± 4.25% (*p* < 0.0001), followed by GCR1 (25.46 ± 3.84%, *p* < 0.0001), PPB1 (22.54 ± 4.07%, *p* = 0004), PPB2 (22.04 ± 4.02%, *p* = 0.007), INH (21.67 ± 3.73%, *p* = 0.003), and BB1 (19.42 ± 4.34, *p* = 0.0029) (Figure 4c). Thus, treatment with these bacterial crude extracts resulted in a significant, time-dependent decrease in the intracellular bacillary survival. To quantify inhibition, the percentage mean inhibition in intracellular bacillary survival induced by each bioactive bacterial crude extract was calculated relative to the untreated-infected control cells over the 24 h treatment period.

The percentage mean inhibition was calculated using the following formula:

$$\text{Percentage mean inhibition}\;(\%)=\frac{\left(\begin{array}{l} {\text{Survival}}_{\text{control},\;\;\text{mean}}-\\ {\text{Survival}}_{\text{treated},\;\;\text{mean}} \end{array}\right)}{\left({\text{Survival}}_{\text{control},\;\;\text{mean}}\right)}\times 100$$

Where ${\text{Survival}}_{\text{control},\;\text{mean}}\;{\text{and Survival}}_{\text{treated},\;\text{mean}}$ represent the mean survival of intracellular bacilli in untreated control cells and in cells treated with bacterial crude extracts, respectively. All bacterial crude extracts demonstrated increasing inhibitory activity over time. Notably, CR1 was the most effective, achieving a 94.4% ± 1.14 mean reduction in intracellular *M. smegmatis* mc^2^155 burden after 24 h treatment (Figure 4d). GCR1, PPB1, BB1, and PPB2 followed closely with mean inhibition rates of 89.18% ± 1.8, 89.10% ± 3.38, 88.18% ± 1.31, and 87.42% ± 3.38, respectively. At 6 h mark, mean inhibition levels were modest, ranging between 10 and 30%, indicating a gradual onset of action. By 12-and 18-h intervals, inhibition exceeded 50% for most extracts, with near-complete inhibition observed after 24 h.

### 3.4 Cultivation of axenic bacterial isolates from CR1 mixed cultures and assessment of their bioactivity

Owing to its low cytotoxicity profile and potent reduction of the intracellular *M. smegmatis* mc^2^155 burden in THP-1-derived macrophages, CR1 was prioritized for the isolation and cultivation of axenic bacterial cultures for subsequent intracellular infection assays and characterization of their bioactive constituents. This was achieved by sub-culturing the CR1 cryopreserved glycerol stock on agar plates using serial dilutions (10^−1^ to 10^−4^), until axenic colonies were obtained. Nine isolates were recovered and cultivated under the same conditions as the mixed bacterial cultures. Secondary metabolites were subsequently extracted from each isolate, and their resulting crude extracts (CR1-1 to CR1-9) were evaluated for antimycobacterial activity following the same protocol used for the mixed bacterial crude extracts. Isolates CR1-1 to CR1-5 exhibited strong antimycobacterial activity, with MIC ranges of between 7.815 to 15.625 μg/mL against *M. smegmatis* mc^2^155 and 3.907 to 7.815 μg/mL against *M. tuberculosis* H37Rv. In comparison, CR1-6 to CR1-9 exhibited moderate activity, with MICs ranges between 62.50 and 125 μg/mL against *M. smegmatis* mc^2^155 and 31.125 to 62.50 μg/mL against *M. tuberculosis* H37Rv (Table 1). In line with the findings from the mixed bacterial crude extracts, axenic extracts that met the susceptibility cut-off criteria (7.815 to 15.625 μg/mL for *M. smegmatis* mc^2^155 and 3.907 to 7.815 μg/mL for *M. tuberculosis* H37Rv) were selected for further intracellular infection screening assays.

**Figure 5:**
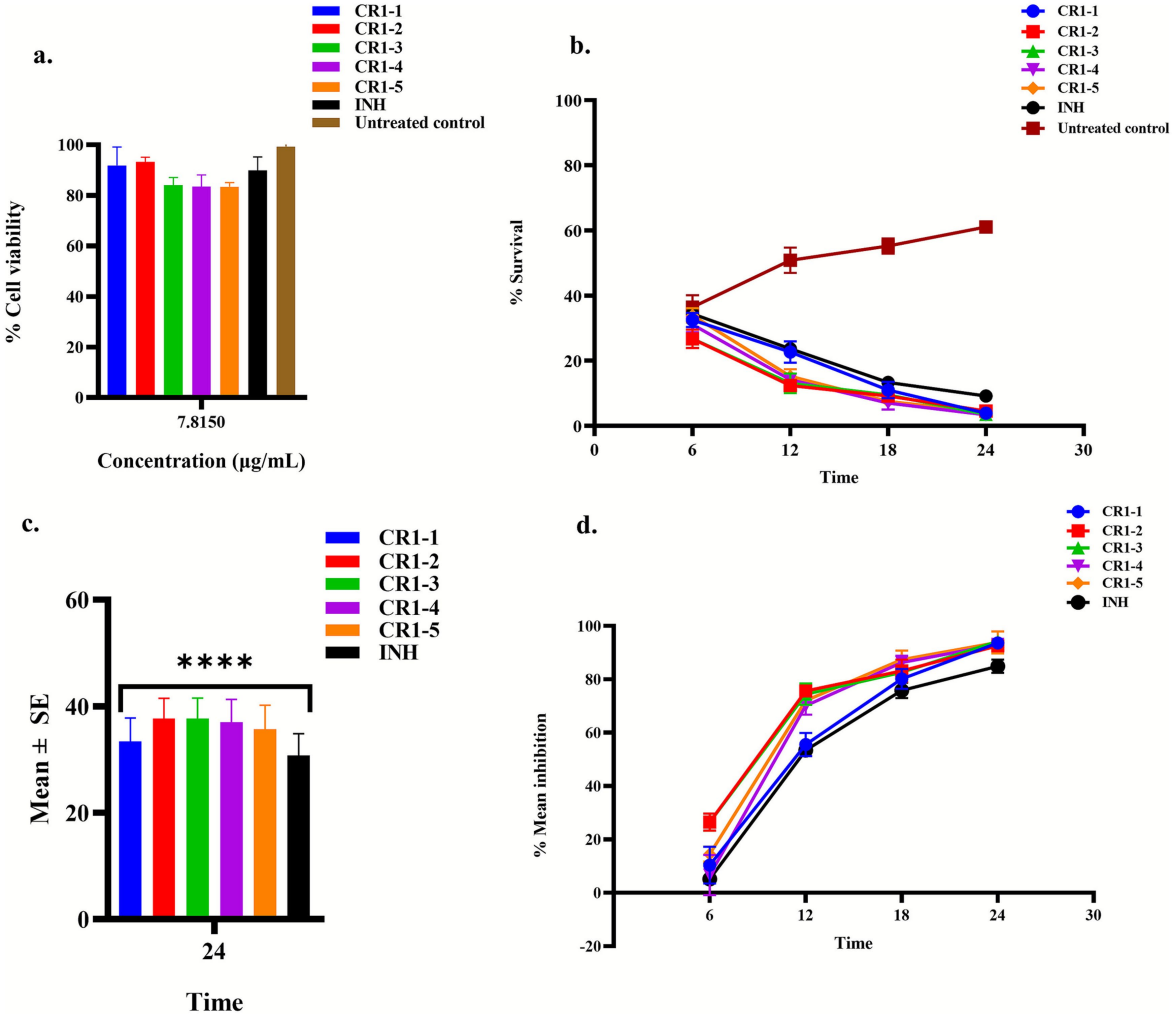
Effect of treatment with axenic bioactive bacterial crude extracts on uninfected and infected THP-1-derived macrophage cells. **(a)** Cytotoxicity (% cell viability) of uninfected THP-1-derived macrophages post-24 h treatment with axenic bacterial crude extracts. Screening was conducted at 7.815 μg/mL, with results represented as mean ± SD in triplicate wells (*n* = 3). **(b)** Percentage survival of intracellular *M. smegmatis* mc^2^155 burden in THP-1-derived macrophages post-treatment with axenic bacterial crude extracts. The results are shown as mean ± SD from triplicate wells (*n* = 3). **(c)** Mean reduction of intracellular *M. smegmatis* mc^2^155 burden in THP-1-derived macrophages post-treatment with axenic bacterial crude extracts. Bars represent SE. Statistical significance was determined using Dunnett’s *post-hoc* multiple tests of comparisons (****p < 0.0001). **(d)** Time-dependent percentage mean inhibition of intracellular *M. smegmatis* mc^2^155 burden in THP-1-derived macrophages post-treatment with the axenic bacterial crude extracts. Data represent mean ± SD of triplicate wells (*n* = 3); error bars indicate replicate variability. Treatment was conducted for 24 h and Isoniazid (INH) was used as a positive control in all experiments.

As a result, extracts CR1-1 to CR1-5 were selected for further evaluation of their efficacy in reducing the intracellular *M. smegmatis* mc^2^155 burden in THP-1-derived macrophages. At 7.815 μg/mL, the selected bioactive bacterial crude extracts exhibited minimal cytotoxicity. Notably, CR1-1 and CR1-2 retained the highest proportion of viable cells at 91.83% and 93.27%, respectively, while CR1-3, CR1-4, and CR1-5 displayed slightly lower survival rates of 83.96%, 83.53%, and 83.37%, respectively (Figure 5a). INH, a reference TB drug, used as a control, maintained a cell viability of 89.87%. Using the same protocol applied for the mixed bacterial crude extracts, we further assessed the ability of these extracts in reducing the intracellular bacilli of *M. smegmatis* mc^2^155 in THP-1-derived macrophages (Figure 5b). Where reduction in intracellular burden was observed, the percentage mean inhibition relative to the untreated control was calculated.

A two-way repeated measures ANOVA revealed significant main effects of treatment and time, along with a significant treatment x time interaction (all *p* < 0.0001), indicating that both factors significantly influenced the reduction of intracellular *M. smegmatis* mc^2^155 burden in THP-1-derived macrophages. Treatments with CR1-1, CR1-2, CR1-3, CR1-4, CR1-5, and INH accounted for 61.09% of the total variance, time for 19.72%, and their interaction for 18.01%. Inter-replicate variability was statistically significant but minimal (*p* = 0.0024), contributing only 0.59% of the total variation. *Post-hoc* Dunnett’s test revealed that the axenic bacterial crude extracts, including INH, significantly reduced intracellular *M. smegmatis* mc^2^155 survival in THP-1-derived macrophages. Notably, CR1-2 and CR1-3 were the most effective, each achieving a mean bacillary reduction of 37.67 ± 3.81% (*p* < 0.0001) and 37.67 ± 3.87% (*p* < 0.0001), respectively (Figure 5c). CR1-4, CR1-5, CR1-1, and INH followed closely with mean bacillary reductions of 36.97 ± 4.31% (*p* < 0.0001), 35.69 ± 4.50% (*p* < 0.0001), 33.39 ± 4.39% (*p* < 0.0001), and 30.75 ± 4.11% (*p* < 0.0001), respectively. To quantify the effects induced by these bacterial crude extracts, percentage mean inhibition was calculated relative to the untreated-infected control cells. All screened bacterial crude extracts demonstrated a consistent, time-dependent decrease of the intracellular *M. smegmatis* mc^2^155 burden in THP-1-derived macrophages. Among these, CR1-3 demonstrated the strongest inhibitory effects, achieving a mean inhibition of 94.00% ± 1.8 post 24 h of treatment (Figure 5d). Following closely were CR1-5, CR1-4, CR1-1, and CR1-2 with mean inhibition rates of 93.80% ± 4.14, 93.62% ± 0.10, 93.09% ± 0.7, and 92.60% ± 1.7, respectively. In comparison, INH achieved a mean inhibition rate of 84.90% ± 2.51, highlighting the superior efficacy of the axenic bacterial crude extracts in reducing the intracellular *M. smegmatis* mc^2^155 burden in THP-1-derived macrophages. By the 6 h mark, mean inhibition rates ranged from 5.5 to 28.3%, indicating a gradual onset of action. By the 12- and 18-h time points, inhibition levels exceeded 50%, with near-complete inhibition observed after 24 h.

### 3.5 Apoptotic and necrotic effects induced by CR-1-derived bacterial crude extracts on THP-1-derived macrophages infected with *M. smegmatis* mc^2^155

**Figure 6:**
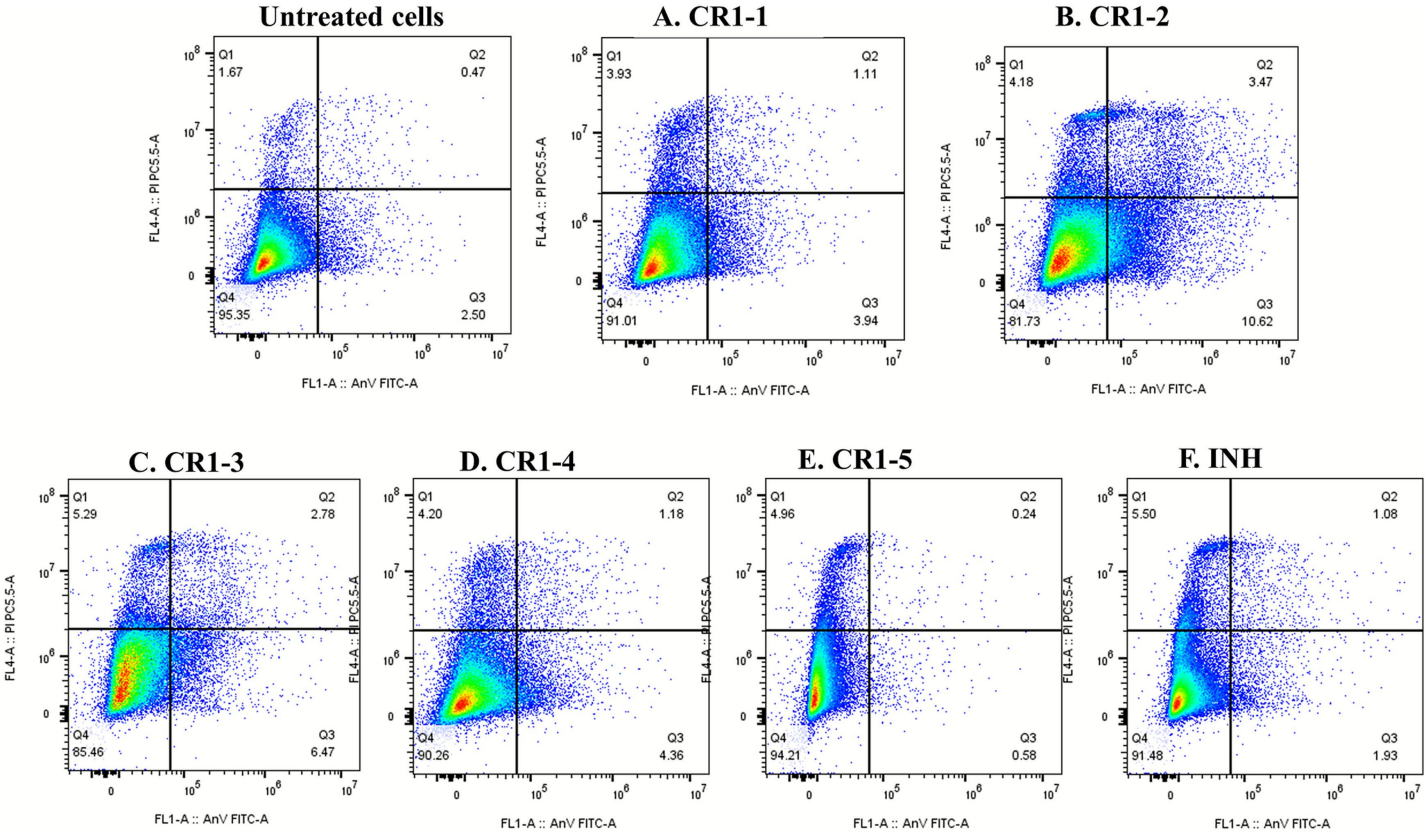
Apoptotic and necrotic cell percentages induced by CR1-1, CR1-2, CR1-3, CR1-4, and CR1-5 axenic bacterial crude extracts on THP-1-derived macrophages infected with *M. smegmatis* mc^2^155. Treatment was conducted for 24 h, and INH was used as a positive control.

Treatment of THP-1-derived macrophages infected with *M. smegmatis* mc^2^155 with CR1-1 to CR1-5 bacterial crude extracts significantly reduced the intracellular bacilli survival. To determine the mode of cell death underlying the reduction of intracellular *M. smegmatis* mc^2^155 burden in THP-1-derived macrophages treated with these bacterial crude extracts, we assessed apoptosis and necrosis using Annexin V and Propidium Iodide (PI) staining, and stained cells were analyzed using flow cytometry. Apoptosis, commonly referred to as programmed cell death, is a regulated process that removes damaged or unwanted cells, helps maintain cellular homeostasis, and protects the host by removing compromised cells during infection (Mustafa et al., 2024). In contrast, necrosis is an unregulated form of cell death that disrupts membrane integrity, causes cell lysis, and triggers inflammation in surrounding tissues (Fink and Cookson, 2005). Annexin V detects phosphatidylserine (PS), a marker of apoptosis, while PI identifies cells with compromised membranes, indicating cell death (Gerke et al., 2024). Cells were categorized as late apoptotic (Annexin V and PI positive, Q2, upper-right quadrant), early apoptotic (Annexin positive, Q3, lower-right quadrant) necrotic (PI positive, Q1, upper-left quadrant) or viable (unstained, Q4, lower-left quadrant) based on fluorescence intensity plots. The gating strategy used for flow cytometric analysis is shown in Supplementary Figure S1. After 24 h of treatment, the bacterial crude extracts CR1-1 to CR1-5 induced comparable levels of apoptosis and necrosis in THP-1-derived macrophages infected with *M. smegmatis* mc^2^155, with apoptosis predominating in most extracts. The observed apoptotic cell percentages relative to the untreated infected control cells were 3.94 ± 0.15% for CR1-1, 10.62 ± 0.07% for CR1-2, 6.47 ± 0.24% for CR1-3, 4.36 ± 0.02% for CR1-4, and 0.58 ± 0.31% for CR1-5, as shown in Figure 6. Moreover, treatment with these extracts also elicited necrotic responses, with necrotic percentages of 3.93 ± 26.05% for CR1-1, 4.18 ± 32.32% for CR1-2, 5.29 ± 42.94% for CR1-3, 4.20 ± 42.19% for CR1-4, and 4.96 ± 29.51% for CR1-5. In comparison, treatment with INH yielded apoptosis rate of 1.93 ± 0.66% and necrotic rate of 5.50 ± 19.77%.

### 3.6 Molecular identification of axenic bacterial isolates

Axenic bacterial isolates obtained from the CR1 mixed cultures were characterized as Gram-negative rods. Taxonomic identification was conducted using 16S rRNA gene sequencing. The CR1-1 isolate was classified within the genus *Marinobacter*, showing 100% sequence identity to *Marinobacter maritimus* (GenBank accession no. KT983995.1). CR1-2 and CR1-3 were closely related to *Psychrobacter celer*, with identities of 99.47% (GenBank accession no. NR_043225.1) and 100% (GenBank accession no. KP058415.1), respectively. CR1-4 was identified as *Pseudomonas benzenivorans* with 100% identity (GenBank accession no. CP073346.1). CR1-5 was affiliated with *Bacillus altitudinis*, showing 99.85% sequence identity (GenBank accession no. CP139561.1). CR1-6 and CR1-7 were both identified as *Paenibacillus glucanolyticus*, with 99.78 and 99.78% sequence identity (GenBank accession no. NR_113748.1), repectively. CR1-8 and CR1-9 were identified as *Bacillus altitudinis*, *Bacillus aerius* or *Bacillus stratosphericus*, with sequence identities of 99.05 and 100%, respectively (GenBank accession no. NR_042337.1/NR_118439.1/NR_042336.1).

### 3.7 Untargeted metabolomic profiling of the axenic bioactive bacterial extracts

The antibacterial and host-modulatory activities observed in these sediment-derived bacterial isolates are likely to result from their ecological adaptations. Many marine microbes coexist with soft-bodied hosts that lack physical defense mechanisms, relying instead on chemically mediated interactions for survival, competition, and symbiosis (Debbab et al., 2010). In this context, the production of secondary metabolites represents a key adaptive strategy (Debbab et al., 2010). In general, marine bacteria are known to produce a wide range of compounds with diverse biological activities, including antibacterial, anti-inflammatory, and host-modulatory properties, making them valuable sources for discovering new bioactive molecules (Debbab et al., 2010). However, the chemical diversity and bioactivity of secondary metabolites produced by marine bacteria derived from South African intertidal marine sediments remain underexplored, particularly regarding their efficacy in the fight against antimycobacterial infections. To address this gap, an untargeted metabolomic profiling of axenic bioactive bacterial crude extracts was conducted using high-resolution LC-QTOF-MS. The analysis revealed substantial metabolic diversity among the isolates, with metabolites displaying mass-to-charge (m/z) ratios ranging from 100 to 1,250. More than 5,000 unique ion features were identified, with each extract contributing 600 to 1,100 distinct or shared molecular entities. Feature alignment and spectral deconvolution confirmed the production of a broad spectrum of secondary metabolites cultivated under standard laboratory conditions. Annotation using public spectral databases and literature-based dereplication enabled the identification of several recurrent metabolites, including tenacibactin B, maremycin D1, and tubercidine, which were consistently detected across all five axenic extracts. A detailed list of annotated metabolites for each extract is provided in the Supplementary Tables S2–S6.

## 4 Discussion

The growing prevalence of antimicrobial resistance underscores the urgent need to identify novel bioactive compounds, particularly from the previously underexplored environments (Ferraz, 2024). Marine ecosystems, especially intertidal marine sediments, harbor metabolically diverse bacterial communities capable of producing structurally unique secondary metabolites with diverse metabolic and antibiotic properties (Lam, 2006), and based on the sampling done in this study, sediment samples from South Africa’s intertidal zones revealed high bacterial diversity with notable antimycobacterial and host-modulatory activities. The primary objective of this study was to identify bacterial isolates that exhibit dual bioactivity: they should not only inhibit mycobacteria but also modulate the host’s immune system. To achieve this, a mixed-culture strategy was employed to cultivate and isolate axenic bacterial cultures with significant antimycobacterial properties.

Initial bioactivity screening was conducted on mixed bacterial crude extracts. Extracts exhibiting the strongest antimycobacterial inhibition were selected for further intracellular infection assays, followed by sub-culturing, isolation and cultivation of axenic bacterial cultures. Among the screened mixed bacterial crude extracts, BB1, PPB1, PPB2, GCR1, and CR1 demonstrated strong antimycobacterial activity against the tested *Mycobacterium* strains and were subsequently selected for further intracellular infection assays. However, an MTT assay was initially carried out to determine the concentration to be used for treating both the uninfected and *M. smegmatis* mc^2^155-infected THP-1-derived macrophages, as well as to assess the potential cytotoxic effects of these extracts. In this regard, most mixed bacterial crude extracts demonstrated lower apparent cytotoxicity at 62.50 μg/mL than at 31.25 μg/mL, indicating a non-monotonic or complex dose–response pattern. Thus, although higher concentrations are generally associated with increased cytotoxicity, complex biological extracts may produce physicochemical constituents that exert distinct and sometimes opposing effects on host-cell metabolic activity, antioxidant defenses, metabolic adaptation, and stress response pathways, thereby promoting adaptive responses at specific concentrations and subsequently resulting in non-monotonic, dose-dependent effects on cell viability similar to those observed in our study. Despite differences in cytotoxicity profiles, all mixed bacterial crude extracts were selected for further intracellular infection assays to assess their efficacy in reducing the intracellular *M. smegmatis* burden in THP-1-derived macrophages. Overall, when compared to untreated control, CR1 demonstrated the lowest percentage survival of *M. smegmatis* mc^2^155 in THP-1-derived macrophages with 28.08 ± 4.25% mean survival (*p* < 0.0001) and 94.4% ± 1.14 mean growth inhibition, thereby justifying its selection for further sub-culturing, isolation, and cultivation of axenic bacterial cultures.

A total of nine axenic bacterial isolates were cultivated from the CR1 mixed cultures, and their resulting bacterial crude extracts, CR1-1 to CR1-9, were evaluated for bioactivity against the same pathogens. Extracts, CR1-1 to CR1-5 exhibited the strongest antimycobacterial activities against *M. smegmatis* mc^2^155 and *M. tuberculosis* H37Rv, and significantly reduced the intracellular *M. smegmatis* mc^2^155 burden in THP-1-derived macrophages, and were further subjected to a flow cytometric analysis using Annexin V and Propidium Iodide (PI) staining to determine the model of cell death at which they were reducing the intracellular bacilli survival in THP-1-derived macrophages with apoptosis and necrosis as our preference mode of cell death. As previously mentioned, apoptosis is a regulated process that removes damaged or unwanted cells in the body, whereas necrosis is an uncontrolled process that leads to membrane loss, cell lysis, and inflammation in host cells. In this context, the flow cytometric analysis revealed that the axenic bacterial crude extracts induced comparable levels of apoptosis and necrosis, with apoptosis predominating in most extracts. These findings suggest that the screened axenic bacterial crude extracts may stimulate inflammatory responses and modulate host-modulatory effects in THP-1-derived macrophages infected with *M. smegmatis* mc^2^155. These observations are consistent with Sousa et al., (2019), who demonstrated that macrophages can induce apoptosis in response to non-virulent mycobacterial strains. Thus, targeting apoptosis or necrosis in macrophages represents a promising host-directed therapy (HDT) strategy that can help improve TB treatment outcomes (Bohsali et al., 2010). Thererefore, it can be hypothesized that restoring apoptotic pathways may allow the host to regain control over the *M. tuberculosis* infection.

Subsequent 16S rRNA gene sequencing of CR1-derived isolates identified five distinct bacterial taxa: *Psychrobacter celer, Marinobacter maritimus*, *Pseudomonas benzenivorans*, *Bacillus altitudinis*, and *Paenibacillus glucanolyticus*. These taxa are recognized for their ability to produce bioactive secondary metabolites, particularly under the extreme conditions’ characteristic of intertidal marine sediments. *Psychrobacter celer* is known to exhibit antimicrobial activity via specific enzymatic pathways, as established in prior studies (Esakkiraj et al., 2022). For example, a cold-active lipase from the *Psychrobacter celer* strain PU3, isolated from the intestine of marine fish *Lutjanus* sp., demonstrated significant antibiofilm activity against *Vibrio parahaemolyticus*, a pathogen associated with aquaculture infections (Esakkiraj et al., 2022). This enzyme inhibited biofilm formation and induced pronounced morphological changes in bacterial cells suggesting disruption of essential structural or regulatory processes (Esakkiraj et al., 2022). Transcriptional analysis indicated substantial downregulation of genes associated with virulence, quorum sensing, and surface attachment (Esakkiraj et al., 2022). Although research interest in *Psychrobacter* species is increasing, particularly regarding their ecological adaptability and probiotic potential, the immune-stimulatory properties of *Psychrobacter celer* remain insufficiently characterized. In the absence of host-modulatory evidence, insights from related species provide relevant context. For instance, Yu et al., (2016) demonstrated that exopolysaccharides from *Psychrobacter* sp. B-3 can enhance the innate immune response of RAW264.7 macrophages by promoting phagocytic activity and inducing pro-inflammatory mediators via TLR/NF-κB signaling pathways. Similarly, Korneev et al., (2014) found that a fully acylated hexaacyl lipid A from *Psychrobacter cryohalolentis* elicited stronger TNF-α responses in bone marrow-derived macrophages. Collectively, these findings, along with the antimycobacterial and immune-modulatory effects observed in this study, indicate that *Psychrobacter* species may produce multifunctional metabolites that target diverse bacterial pathogens and enhance host immune cell function during infection.

Although the antimicrobial profile of *Pseudomonas benzenivorans* remains uncharacterized, other species within the *Pseudomonas* genus are established producers of bioactive metabolites (Isnansetyo and Kamei, 2009). For instance, two phenolic siderophores, Pseudobactin UIAU-6B-1 and UIAU-6B-2, isolated from *Pseudomonas* sp. UIAU-6B obtained from oil-contaminated marine sediments in the Niger Delta of Nigeria, demonstrated significant antibacterial activity against *Enterococcus faecium*, *Staphylococcus aureus*, and *M. smegmatis* Mc^2^155, with MICs of 8, 32, and 64 μg/mL for Pseudobactin UIAU-6B-1, and 32, 64, and 128 μg/mL for Pseudobactin UIAU-6B-2, respectively (Oluwabusola et al., 2021). In addition to their antibacterial properties, *Pseudomonas* species also display immune-modulatory activities (Moser et al., 2021; Skindersoe et al., 2009; Thomas et al., 2006). For example, soil-derived lipopolysaccharides (LPS) from *Pseudomonas* sp. PNK-O4r have been shown to trigger pro-inflammatory responses in RAW264.7 macrophages through the activation of TLR4/NF-κB and MAPK signaling pathways (Thomas et al., 2006). Similarly, *Bacillus* species, particularly *Bacillus altitudinis,* are recognized for their ability to produce secondary metabolites that posesses both antibacterial and immune-modulatory properties (Abednego and Silago, 2023). For example, *Bacillus altitudinis*/*pumilus* complex (strain S9D), obtained from Mwanza, Tanzania, exhibited broad-spectrum antibacterial activity against clinically relevant pathogens, including *Streptococcus pyogenes*, coagulase-negative *Staphylococcus*, and methicillin-susceptible *Staphylococcus aureus* (Abednego and Silago, 2023). Moreover, *Bacillus altitudinis* strain GUHC-03, isolated from coastal sediments of Mexico, displayed antagonistic effects against *Vibrio parahaemolyticus* and *Staphylococcus aureus*, with bioactivity attributed to extracellular metabolites (Galaviz-Silva et al., 2018). Additionally, this strain produced moderate biofilms, suggesting that sessile growth may enhance the production of secondary metabolites (Galaviz-Silva et al., 2018). Furthermore, *Bacillus altitudinis* strain 1.4, isolated from the Brazilian wetlands, exhibited remarkable host modulatory capabilities by rapidly increasing TLR-2 and TNF-*α* expression while promoting anti-inflammatory IL-10 and IL-4 cytokines in murine J774 macrophages (Jankoski et al., 2024). Notably, both viable and inactivated cells were capable of triggering immune responses, indicating that cell viability is not essential for activation (Jankoski et al., 2024). These findings underscore the potential of *Bacillus* species in regulating immune responses and supporting immune homeostasis.

The antibacterial and host-modulatory effects of *Marinobacter* species are intricately linked with their production of biosurfactants and exopolysaccharides, particularly within the contexts of *Marinobacter litoralis* and *Marinobacter nauticus*. For instance, a biosurfactant derived from *Marinobacter litoralis* MB15, isolated from the seawater of Roch Beach in Pondicherry, India, demonstrated complete inhibition of *Streptococcus pyogenes* and *Klebsiella pneumoniae* at 25 μg/mL (Haque et al., 2020). Additionally, full inhibition of *Staphylococcus aureus*, *Candida albicans*, and *Vibrio parahaemolyticus* was observed at 50 μg/mL (Haque et al., 2020). Furthermore, Exopolysaccharides (EPS) derived from *Marinobacter nauticus* GH, obtained from the Red Sea seawater near Sharm El-Sheikh, Egypt, were found to stimulate immune responses in both murine and human THP-1 macrophages (Abdel-Razik et al., 2024). In human THP-1 macrophages, the EPS induced dose-dependent secretion of pro-inflammatory cytokines, including IL-1β, IL-6, and TNF-α, along with the anti-inflammatory cytokine IL-10 (Abdel-Razik et al., 2024). In murine RAW 264.7 macrophages (J774 cell line), the EPS facilitated the secretion of TNF-α and IL-10 while promoting cell proliferation without inducing cytotoxicity (Abdel-Razik et al., 2024). Although the direct pathways by which these bacterial extracts promote *M. smegmatis* mc^2^155 killing in THP-1-derived macrophages were not investigated in this study, the observed antimicrobial and host-modulatory activities induced by these isolates suggest that members of these bacterial taxa may produce secondary metabolites that function through multiple mechanisms, integrating direct bacterial effects with modulation of host immune responses.

While this study is the first to report on the antimycobacterial and host-modulatory activities of secondary metabolites produced by bacterial isolates from False Bay intertidal marine sediments, an intriguing observation was the distinct difference in bioactivity between crude extracts from mixed bacterial cultures and those from axenic cultures. Notably, crude extracts from axenic cultures demonstrated stronger antimycobacterial activity than those from mixed cultures, underscoring the influence of microbial interactions on metabolic production (Yan et al., 2023). The variation in bioactivity may result from interactions occurring in co-cultures, such as competition or cross regulation, which can suppress or disrupt the production of secondary metabolites by dominant species, thereby reducing their overall bioactivity (Yan et al., 2023; LaSarre and Federle, 2013; Sanders et al., 2023). Thus, the production of bioactive molecules produced by mixed bacterial cultures may be diluted within the broader metabolic output of the microbial community, thereby diminishing their apparent efficacy (Ogofure and Green, 2025). Furthermore, these interactions often promote ecological balance rather than competitive stress, leading to a reduction in metabolic production when compared to systems with single isolates (Yu et al., 2024; Selegato and Castro-Gamboa, 2023; Peng et al., 2021). Our findings corroborate previous research indicating that interspecies co-culturing often suppresses the production of secondary metabolite (Hansen et al., 2022). For example, a study conducted by Westhoff et al., (2021) demonstrated that while one-third of co-culturing interactions resulted in the production of antibiotics, nearly half exhibited suppression, particularly under nutrient-limited conditions (Westhoff et al., 2021). Induction of secondary metabolites was common among closely related isolates or those with shared biosynthetic clusters, suggesting that metabolite production may be influenced by signaling molecules rather than direct antagonism (Westhoff et al., 2021). Conversely, the notable frequency of suppression implies that co-culturing may serve as an energy-conservation strategy or developmental adaptation, underscoring the importance of ecological context in microbial metabolism (Westhoff et al., 2021). Similarly, Svendsen et al. (2024) revealed that coexisting antibiotic-producing bacteria, *Phaeobacter* sp. and *Pseudoalteromonas piscicida*, reduced rather than increasing the tropodithietic acid (TDA) production in *Phaebacter*. *Pseudoalteromonas piscicida* suppressed the expression of the TDA biosynthetic gene cluster in *Phaeobacter* sp., resulting ina marked decrease in TDA production (Svendsen et al., 2024). Additionally, Chevrette et al., (2022) found that within a three-rhizosphere model community, *Pseudomonas koreensis* significantly altered the expression of petrobactin biosynthetic gene clusters in *Bacillus cereus*. Notably, these changes occurred exclusively within the full three-member consortium, with no variation in pairwise interactions (Chevrette et al., 2022). Collectively, these findings demonstrate that although microbial co-culturing can sometimes enhance secondary metabolite production, it does not invariably result in increased bioactivity. Factors such as transcriptional downregulation, regulatory interference, or species dominance may suppress the synthesis of secondary metabolites, thereby reducing their overall bioactivity (Pardo-Esté et al., 2024; Chen et al., 2024; Gorter et al., 2020). This highlights the complexity of microbial consortia, which exhibit non-additive interactions and exert significant influence on the ecological functions of microbial communities (Haruta and Yamamoto, 2018; Jaiswal et al., 2025; Abrudan et al., 2015).

## 5 Conclusions and future recommendations

Macrophages play a central role in the pathophysiology of TB, serving as the primary host cells for *M. tuberculosis*. However, the pathogen has evolved mechanisms to evade and manipulate the host’s immune defenses, enabling its survival and replication within macrophages, highlighting the need for complementary therapies that reduce dependence on high-dose, pathogen-targeted treatments and limit the emergence of drug-resistant strains. The present study investigated South African intertidal marine sediments as sources of bacteria that produce bioactive secondary metabolites with antimycobacterial and host-modulatory properties. Bioassay-guided screening of crude mixed bacterial cultures enabled the cultivation and identification of axenic bacterial isolates exhibiting significant antimycobacterial and host-modulatory activities. A fundamental aspect of this research involved using *M. smegmatis* mc^2^155 as a surrogate model for initial macrophage infection assays. While *M. smegmatis* mc^2^155 is widely used due to its rapid growth and physiological similarities to *M. tuberculosis*, it does not fully replicate the pathogenic characteristics or intracellular behavior of *M. tuberculosis*, which constitutes a significant limitation. Therefore, subsequent research will aim to assess the efficacy of active bacterial crude extracts against *M. tuberculosis* H37Rv-infected THP-1 macrophages, thereby improving physiological relevance and providing a more accurate evaluation of therapeutic potential. Although preliminary results using crude bacterial extracts suggest considerable therapeutic promise, reliance on crude extracts remains a limitation, as the complexity of these mixtures may obscure the activity of individual metabolites. To address this, future research will prioritize the fractionation, purification, and structural characterization of the active compounds to identify novel chemical entities and clarify the mechanisms underlying the observed biological effects. In conclusion, this study advances understanding of the biodiversity and distribution of marine bacteria from intertidal sediments in False Bay, South Africa, and underscores their potential as sources of bioactive metabolites with antimycobacterial and host-modulatory properties. Further investigations using purified metabolites and pathogenic models of *M. tuberculosis* are expected to enhance knowledge of their therapeutic potential and facilitate the development of novel antimycobacterial agents with host-modulatory effects.

## Data Availability

The datasets used and analyses during the current study are available from the corresponding authors upon reasonable request.

## Footnotes

1
https://inqababiotec.co.za/

2
https://blast.ncbi.nlm.nih.gov/Blast.cgi

3
http://prime.psc.riken.jp/compms/msfinder/main.html
