# Supplementary material for: Antimycobacterial activity of intertidal sediment-derived bacteria from False Bay, South Africa
Source: Front Microbiol. 2026 Jan 15;16:1745248. doi: 10.3389/fmicb.2025.1745248 (PMC12852442; doi:10.3389/fmicb.2025.1745248)
Supplement: Supplementary file 1 [file Data_Sheet_1.pdf]

Supplementary Material

Supplementary methods

2.6. Sample preparation, DNA extraction, and 16S rRNA sequencing of mixed bioactive bacterial cultures

2.6.1. The following protocol describes in detail sample preparation, DNA extraction, and 16S rRNA sequencing of the bioactive mixed bacterial cultures

For each bioactive sample, 50 mL of bacterial culture was prepared by inoculating mixed bacterial colonies into a 50 mL LB broth media and sent for DNA extraction and full-length 16S rRNA gene sequencing by the Inqaba biotechnology industry (Pretoria, South Africa). https://inqababiotec.co.za/. Briefly, the bacterial sample was added to a ZR BashingBead^{TM} Lysis tubes, to which 750 µL ZymoBIOMICS^{TM} lysis solution was then added and capped tightly. Samples were lysed using an optimized beat beating condition. The ZR BashingBead^{TM} lysis tubes were centrifuged at 10 000 X g for 1 min. Following centrifugation, 400 µL of the supernatant was transferred to the Zymo-Spin^{TM} III-F Filter in a collection tube and centrifuged at 8000 X g for 1 min. The Zymo-Spin^{TM} III-F Filter was discarded, and 1200 µL of ZymoBIOMICS^{TM} DNA Binding Buffer was added to the filtrate in the collection tube and gently mixed. A total of 800 µL of the mixed solution was transferred to a Zymo-Spin^{TM} IICR Column in a collection cube and centrifuged at 10000 X g for 1 min. The flow through was discarded and 800 µL of the mixed solution was transferred back into a Zymo-Spin^{TM} IICR Column and centrifuged at 10000 X g for 1 min. Thereafter, 400 µL of ZymoBIOMICS^{TM} DNA wash Buffer 1 was added to the Zymo-Spin IICR Column in a new column tube and centrifuged at 10000 X g for 1 min.

After centrifugation, the flow through was discarded and 700 µL of the ZymoBIOMICS^{TM} DNA wash Buffer 2 was added and centrifuged at 10000 X g for 1 min. The flow through was discarded, and 200 µL of ZymoBIOMICS^{TM} DNA wash Buffer 2 was added to the Zymo-Spin IICR Column in a collection tube and centrifuged at 10000 X g for 1 min. The Zymo-Spin IICR Column was transferred to a clean 1.5 mL microcentrifuge tube, and 100 µL of ZymoBIOMICS^{TM} DNase-free water was added to the column matrix, incubated for 1 min, and centrifuged at 10000 X g for 1 min. A Zymo-Spin^{TM} III-HCR filter was then paced in a new collection tube and 600 µL of ZymoBIOMICS^{TM} HRC Prep Solution

was added, and centrifuged at 8000 X g for 3 min. The eluted DNA was then transferred to a prepared Zymo-Spin™ III-HRC filter in a clean 1.5 mL microcentrifuge tube and centrifuged at 16000 X g for 3 min. The filtered DNA was then sequenced using the Sequel lle by PacBio (www.pacb.com). Raw sub-reads were then processed through the SMRTlink (v11.0) Circular Consensus Sequences (CSS) algorithm to produce highly accurate reads (>QV40). For quality control assessment and taxonomic classification, highly reads were processed through the DADA2 (https://benjjneb.github.io/dada2/index.html), and qiime2 (https://docs.qiime2.org/2021.11/). The Illumina-sequenced data was then transformed into an amplicon sequence variant (ASV) table following the DADA2 pipeline.

Table S1. Geographic coordinates for each sampling site

**Collection site**	**Site-coordinates**	**Site code**	**No. of sediments samples collected**
Buffles Bay beach	34°.19'04.4"S 18°27'39.2"E	BB	2
Castle Rock beach	-34°.14'21.06"S 18°28'36.66"E	CR	2
Glencairn beach	33°.43'27.0"S 18°26'31.5"E	GCR	2
Cape of Good Hop	34°.21'24.6"S 18°28'25.3"E	CGH	1
Diaz beach	34°.21'13"S 18°28'55"E	DB	2
Pappies bank	34.3540° S, 18.4720° E	PPB	2
Grotto bay	33.5056° S, 18.3150° E	GB	2
Lagoon beach	33.8907° S, 18.4826° E	LB	2
Melkbostrand beach	33°43'27.0"S 18°26'31.5"E	MBS	2

Supplementary Figures

Supplementary Figure S1. Gating strategy for apoptotic and necrotic cell analysis

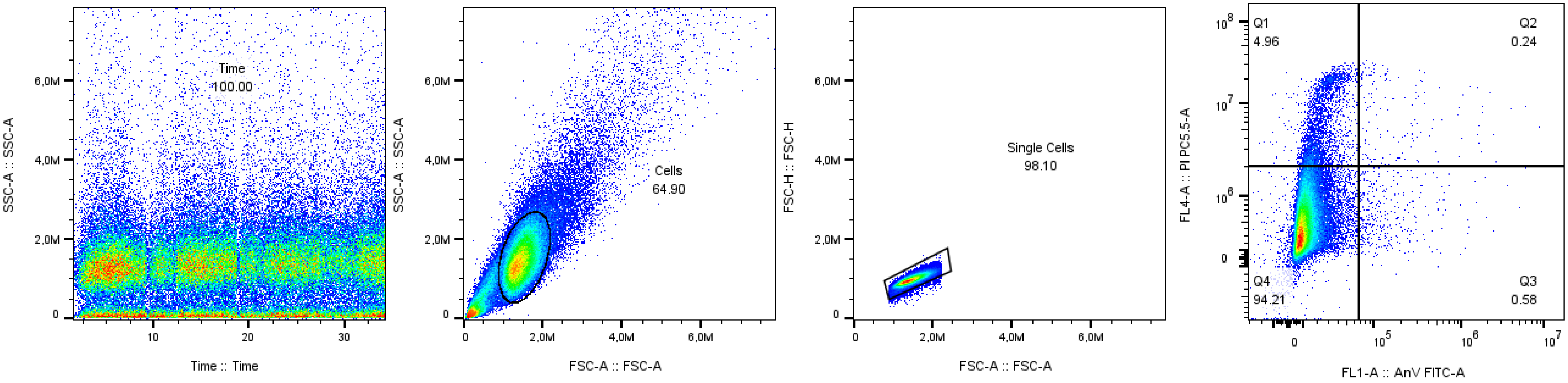

Figure S1. Gating strategy for the flow cytometric analysis of apoptotic and necrotic cells. Representative dot plots showing gating hierarchy used to distinguish between viable (Q4), early apoptotic (Q3), late apoptotic (Q2) and necrotic cells (Q1) post 24 h treatment with axenic bacterial crude extracts. Cells were first gated based on forward scatter (FSC) and side scatter (SSC) to exclude debris and doublets. Subsequent quadrants were defined using Annexin V-FITC and propidium iodide (PI) fluorescence: Q4 (Annexin V^-/PI^-): viable cells; Q3 (Annexin V^+/PI^-): early apoptotic cells; Q2 (Annexin V^+/PI^+): late apoptotic cells; and Q1 (V-/PI+): primary necrotic cells. Gates were established using untreated control cells and single-stained compensation controls.

Supplementary Tables S2-S6

Supplementary Table S2. LC/MS metabolite profile of CR1-1 bacterial isolate

R/T. (Min)	Meas.(M/Z)	Calc.(m/z)	Error.ppm	Formulae	Adducts	Compound name	Ontology
2.502	266.1877	265.179	5.34	$C_{14}H_{23}N_3O_2$	[M+H] +	Streptopyrazinone C	Pyrazines
7.908	267.1105	266.1015	6.45	$C_{11}H_{14}N_4O_4$	[M+H] +	Tubercidine	Pyrrolopyrimidine nucleosides and nucleotides
2.332	267.1105	266.1015	6.45	$C_{11}H_{14}N_4O_4$	[M+H] +	Coaristeromycin	Nucleoside and nucleotide analogues
3.682	267.1105	266.1015	6.45	$C_{11}H_{14}N_4O_4$	[M+H] +	dehydrodeoxycoformycin	Imidazodiazepines
3.261	267.111	266.1055	-6.64	$C_{16}H_{14}N_2O_2$	[M+H] +	Nannozinone B	Pyrrolopyrazines
4.335	269.1481	268.1423	-5.48	$C_{13}H_{20}N_2O_4$	[M+H] +	Cladosin F	Cyclic carboximidic acids
4.573	162.0922	161.0841	5.08	$C_{10}H_{11}NO$	[M+H] +	Streptazone C	Tetrahydropyridines
3.493	269.1627	268.1576	-8.08	$C_{17}H_{20}N_2O$	[M+H] +	1,3-Diphenethylurea	Benzene and substituted derivatives
2.743	270.2805	269.2719	4.89	$C_{17}H_{35}NO$	[M+H] +	Bacillamidin G	Carboximidic acids
2.893	272.1614	271.1532	3.39	$C_{12}H_{21}N_3O_4$	[M+H] +	Vazabitide A	Peptides
4.243	272.163	271.1572	-5.42	$C_{17}H_{21}NO_2$	[M+H] +	Erythrozeylanine B	Cinnamic acid esters
2.874	272.2133	271.2048	4.49	$C_{17}H_{25}N_3$	[M+H] +	Mirabilin A	Quinazolines
2.308	273.1236	272.1161	0.82	$C_{15}H_{16}N_2O_3$	[M+H] +	Limazepine E	1,4-benzodiazepines
8.057	273.1486	272.1412	0.45	$C_{17}H_{20}O_3$	[M+H] +	Haterumadysin B	Indanes
2.844	273.16	272.1525	0.82	$C_{16}H_{20}N_2O_2$	[M+H] +	L-tryptophan	Indolyl carboxylic acids and derivatives
3.761	273.16	272.1525	0.82	$C_{16}H_{20}N_2O_2$	[M+H] +	Usabamycin A	1,4-benzodiazepines
3.991	273.1812	272.1736	1.18	$C_{13}H_{24}N_2O_4$	[M+H] +	Elaiomycin D	Azoxy compounds
5.922	275.1025	274.0954	-0.63	$C_{14}H_{14}N_2O_4$	[M+H] +	Venezueline B	Benzoxazines
2.502	276.1085	275.1018	-2.08	$C_{12}H_{13}N_5O_3$	[M+H] +	5'-Deoxytoyocamycin	Glycosylamines
4.192	277.1552	276.1474	1.89	$C_{15}H_{20}N_2O_3$	[M+H] +	Cyclo-(Leu-Tyr)	Alpha amino acids and derivatives
3.85	279.121	278.1154	-6.00	$C_{15}H_{18}O_5$	[M+H] +	Pentalenolactone F	Delta valerolactones
3.065	279.1695	278.163	-2.77	$C_{15}H_{22}N_2O_3$	[M+H] +	Terragine C	Phenylacetamides
5.353	279.1708	278.163	1.87	$C_{15}H_{22}N_2O_3$	[M+H] +	Corallorazine A	Alpha amino acids and derivatives
2.676	280.1555	279.1471	4.01	$C_{15}H_{21}NO_4$	[M+H] +	Salinosporamide B	Alpha amino acid esters
2.425	281.1152	280.1099	-7.0	$C_{18}H_{16}O_3$	[M+H] +	Xenofuranone A	Butenolides
13.875	281.1502	280.1423	2.21	$C_{14}H_{20}N_2O_4$	[M+H] +	Bohemamine E	Pyrrolizines
12.168	282.1705	281.1627	1.85	$C_{15}H_{23}NO_4$	[M+H] +	Secocycloheximide A	Tetrahydropyridines
12.175	283.1055	282.0964	6.44	$C_{11}H_{14}N_4O_5$	[M+H] +	dehydrocoformycin	Glycosylamines
12.175	283.1055	282.0964	6.44	$C_{11}H_{14}N_4O_5$	[M+H] +	Deoxynybomycin	Hydroquinolones

12.175	284.1407	283.1321	4.65	$C_{16}H_{17}N_3O_2$	[M+H] +	Tryptophandehydrobutyrine diketopiperazine	Alpha amino acids and derivatives
12.175	284.1859	283.1784	0.78	$C_{15}H_{25}NO_4$	[M+H] +	Aerocyanidin	Long-chain fatty acids
12.175	284.2234	283.2147	5.01	$C_{16}H_{29}NO_3$	[M+H] +	Tumonoic acid D	Proline and derivatives
12.182	285.2441	284.2351	6.04	$C_{17}H_{32}O_3$	[M+H] +	Tanikolide	Long-chain fatty alcohols
12.175	286.1785	285.1729	-5.85	$C_{18}H_{23}NO_2$	[M+H] +	Dienomycin B	Styrenes
12.175	169.0978	168.0898776	3.82	$C_8H_{12}N_2O_2$	[M+H] +	cyclo(DeltaAla-L-Val)	Alpha amino acids and derivatives
12.175	287.1392	286.1317424	0.63	$C_{16}H_{18}N_2O_3$	[M+H] +	Spinamycin	Phenylhydrazines
3.198	289.143	288.1361591	-1.50	$C_{17}H_{20}O_4$	[M+H] +	Phaeochromycin D	Chromones
3.843	293.148	292.1423071	-5.40	$C_{15}H_{20}N_2O_4$	[M+H] +	Epiderstatin	Piperidinediones
4.671	296.1615	295.1532062	3.43	$C_{14}H_{21}N_3O_4$	[M+H] +	Streptcytosine J	Hydroxypyrimidines
2.502	298.2737	297.2667794	-1.19	$C_{18}H_{35}NO_2$	[M+H] +	Streptoaminal	Azaspirodecane derivatives
13.981	299.1012	298.0953569	-4.79	$C_{16}H_{14}N_2O_4$	[M+H] +	nybomycin	4-quinolinemethanols
2.395	300.1195	299.1117353	1.62	$C_{12}H_{17}N_3O_6$	[M+H] +	Nagstatin	Imidazopyridines
3.657	301.1927	300.183778	5.46	$C_{18}H_{24}N_2O_2$	[M+H] +	Trichostatin RK	Alkyl-phenylketones
2.676	304.1099	303.1007767	6.07	$C_{18}H_{13}N_3O_2$	[M+H] +	Mansouramycin D	Isoquinoline quinones
4.26	304.153	303.1443728	4.44	$C_{13}H_{17}N_7O_2$	[M+H] +	Kikumycin A	Proline and derivatives
2.874	307.167	306.1579572	5.75	$C_{16}H_{22}N_2O_4$	[M+H] +	Phthoxazolin B	Oxazoles
4.449	308.1473	307.1419728	-6.32	$C_{16}H_{21}NO_5$	[M+H] +	Bacillcoumacin A	2-benzopyrans
3.838	309.2036	308.1987594	-7.87	$C_{18}H_{28}O_4$	[M+H] +	Albocycline	Macrolides and analogues
3.472	310.1768	309.1688562	2.15	$C_{15}H_{23}N_3O_4$	[M+H] +	Streptcytosine E	N-arylamides
3.706	173.0926	172.0847922	3.07	$C_7H_{12}N_2O_3$	[M+H] +	Valanimycin	Alpha amino acids and derivatives
2.135	313.2272	312.2201635	-0.76	$C_{20}H_{28}N_2O$	[M+H] +	Marinopyrazinone A	Pyrazines
2.196	319.1809	318.1732133	1.28	$C_{21}H_{22}N_2O$	[M+H] +	Hapalindolinone B	Sesquiterpenoids
6.981	320.2228	319.2147438	2.43	$C_{19}H_{29}NO_3$	[M+H] +	Cinnabaramide C	Alpha amino acids and derivatives
7.039	322.1768	321.1688562	2.07	$C_{16}H_{23}N_3O_4$	[M+H] +	Streptcytosine M	Hydroxypyrimidines
13.981	324.1913	323.1845	-1.46	$C_{16}H_{25}N_3O_4$	[M+H] +	Streptcytosine K	Hydroxypyrimidines
2.455	325.0848	324.0779926	-1.44	$C_{14}H_{16}N_2O_5S$	[M+H] +	Asparenomycin C	Carbapenems
7.913	325.1058	324.0997736	-3.84	$C_{19}H_{16}O_5$	[M+H] +	2,3-Dehydro-UWM6	Phenanthrenes and derivatives
7.432	325.12	324.111007	5.28	$C_{18}H_{16}N_2O_4$	[M+H] +	mycocyclosin	Macrolactams
5.708	325.2007	324.193674	-0.76	$C_{18}H_{28}O_5$	[M+H] +	Linieodolide B	Lineolic acids and derivatives
2.19	325.2007	324.193674	-0.76	$C_{18}H_{28}O_5$	[M+H] +	Albocycline M-4	Macrolides and analogues
2.732	325.2007	324.193674	-0.76	$C_{18}H_{28}O_5$	[M+H] +	Albocycline M-5	Macrolides and analogues
2.388	325.2007	324.193674	-0.76	$C_{18}H_{28}O_5$	[M+H] +	Albocycline M-2	Macrolides and analogues
2.528	325.2007	324.193674	-0.76	$C_{18}H_{28}O_5$	[M+H] +	Albocycline M-1	Macrolides and analogues
2.502	326.3041	325.2980795	-3.84	$C_{20}H_{39}NO_2$	[M+H] +	Semiplenamide C	N-acyl amines

7.908	327.1334	326.1266571	-1.62	$C_{18}H_{18}N_2O_4$	[M+H] +	cyclo(tyrosyl-tyrosyl)	Alpha amino acids and derivatives
2.332	329.1365	328.1310737	-5.61	$C_{19}H_{20}O_5$	[M+H] +	Ochracenomicin C	Hydroxyanthraquinones
3.682	330.1437	329.1375561	-3.42	$C_{17}H_{19}N_3O_4$	[M+H] +	Mazethramycin	1,4-benzodiazepines
3.261	330.168	329.1586855	6.17	$C_{14}H_{23}N_3O_6$	[M+H] +	Valclavam	Dipeptides
4.335	331.1674	330.1579572	6.54	$C_{18}H_{22}N_2O_4$	[M+H] +	Quinocarcin	Tetrahydroisoquinolines
4.573	333.1959	332.1888634	-0.71	$C_{22}H_{24}N_2O$	[M+H] +	Lavanducyanin	Phenazines and derivatives
3.493	335.1403	334.1317424	3.82	$C_{20}H_{18}N_2O_3$	[M+H] +	Isomethoxyneihumicin	Methoxypyrazines
2.743	336.1425	335.1368874	-4.94	$C_{17}H_{21}NO_6$	[M+H] +	Microansamycin H	Benzoxepines
2.893	337.1054	336.0997736	-4.89	$C_{20}H_{16}O_5$	[M+H] +	Hatomarubigin B	Angucyclines
4.243	337.163	336.1572885	-4.64	$C_{18}H_{24}O_6$	[M+H] +	Dihydrotetrodecamycin	Butenolides
2.874	340.2255	339.2198292	-4.71	$C_{22}H_{29}NO_2$	[M+H] +	Antartin	Sesquiterpenoids
2.308	340.2353	339.2270398	2.89	$C_{16}H_{29}N_5O_3$	[M+H] +	Sperabillin B	Beta amino acids and derivatives
8.057	345.1123	344.1042074	2.36	$C_{14}H_{20}N_2O_6S$	[M+H] +	Carpetimycin C	Thienamycins
2.844	345.1773	344.1695845	1.27	$C_{14}H_{24}N_4O_6$	[M+H] +	rhodotorulic acid	Alpha amino acids and derivatives
3.761	347.1799	346.1740012	-3.96	$C_{15}H_{26}N_2O_7$	[M+H] +	Spenolimycin	1,4-dioxanes
3.991	384.1566	383.1481208	3.13	$C_{20}H_{21}N_3O_5$	[M+H] +	Pelagiomicin B	Phenazines and derivatives
5.922	384.265	383.2572773	1.16	$C_{23}H_{33}N_3O_2$	[M+H] +	Methylpendolmycin	Alpha amino acid amides
2.502	384.2759	383.2671733	3.77	$C_{21}H_{37}NO_5$	[M+H] +	Tumonoic acid F	Proline and derivatives
4.192	393.1816	392.1736072	1.82	$C_{23}H_{24}N_2O_4$	[M+H] +	Endophenazine F1	Phenazines and derivatives
3.85	393.2379	392.2311221	-1.26	$C_{21}H_{32}N_2O_5$	[M+H] +	Cyrmenin B1	Peptides
3.065	397.2102	396.2049074	-4.99	$C_{23}H_{28}N_2O_4$	[M+H] +	Diazaquinomycin E	Quinoline quinones
5.353	398.2426	397.2365419	-3.05	$C_{23}H_{31}N_3O_3$	[M+H] +	Cyclomarazine A	Alpha amino acids and derivatives
2.676	404.2863	403.2794693	-1.10	$C_{18}H_{37}N_5O_5$	[M+H] +	Istamycin C	Aminocyclitol glycosides
2.425	406.1839	405.1760668	1.37	$C_{17}H_{23}N_7O_5$	[M+H] +	Cytomycin	Pyrimidones
13.875	406.2382	405.2303939	1.30	$C_{26}H_{31}NO_3$	[M+H] +	Lavanduquinocin	Carbazoles
12.168	411.1894	410.1841719	-4.98	$C_{23}H_{26}N_2O_5$	[M+H] +	Streptophenazine C	Phenazines and derivatives
12.175	413.1848	412.1746699	6.90	$C_{21}H_{24}N_4O_5$	[M+H] +	Cyanocycline B	Isoquinoline quinones
12.175	414.1274	413.1222999	-5.25	$C_{20}H_{19}N_3O_7$	[M+H] +	Proximicin B	2-heteroaryl carboxamides
12.175	414.2728	413.267842	-5.59	$C_{24}H_{35}N_3O_3$	[M+H] +	Quinocinnolinomycin D	Aryl ketones
12.175	426.1903	425.1838376	-1.90	$C_{24}H_{27}NO_6$	[M+H] +	Nisamycin	Medium-chain fatty acids
12.175	426.1903	425.1838376	-1.90	$C_{24}H_{27}NO_6$	[M+H] +	platencin	Medium-chain fatty acids
12.182	430.2318	429.2263711	-4.29	$C_{23}H_{31}N_3O_5$	[M+H] +	Sibanomicin	Aminoglycosides
12.175	437.192	436.1845659	0.36	$C_{21}H_{28}N_2O_8$	[M+H] +	Deisovalerylblastmycin	Acylaminobenzoic acid and derivatives
12.175	444.1988	443.1944023	-6.47	$C_{24}H_{29}NO_7$	[M+H] +	platensin A4	Acylaminobenzoic acid and derivatives
12.175	446.2912	445.2828233	2.46	$C_{26}H_{39}NO_5$	[M+H] +	Piericidin B1 N-oxide	Sesquiterpenoids

12.175	449.2705	448.2645475	-2.94	$C_{18}H_{36}N_6O_7$	[M+H] +	Fortimicin C	Aminocyclitol glycosides
3.198	450.2901	449.2849486	-4.71	$C_{19}H_{39}N_5O_7$	[M+H] +	gentamycin C1a	Aminocyclitol glycosides
2.304	452.325	451.3198776	-4.76	$C_{28}H_{41}N_3O_2$	[M+H] +	Olivoretin D	Alpha amino acid amides
14.164	453.2877	452.278741	3.71	$C_{26}H_{36}N_4O_3$	[M+H] +	Antiostatin B3	Carbazoles
4.671	454.2942	453.2879087	-2.16	$C_{28}H_{39}NO_4$	[M+H] +	Aureoverticillactam	Cyclic carboximidic acids
3.205	455.232	454.2274933	-6.08	$C_{17}H_{34}N_4O_{10}$	[M+H] +	Hetangmycin	4,5-disubstituted 2-deoxystreptamines
2.502	456.2768	455.2671733	5.15	$C_{27}H_{37}NO_5$	[M+H] +	Mirilactam A	Macrolactams
13.981	460.212	459.2077609	-6.59	$C_{17}H_{29}N_7O_8$	[M+H] +	Glycinothricin	Glycosylamines
2.395	476.3077	475.3005987	-0.36	$C_{21}H_{41}N_5O_7$	[M+H] +	Netilmicin	Aminocyclitol glycosides
3.657	481.2863	480.2795289	-1.04	$C_{20}H_{40}N_4O_9$	[M+H] +	Combimicin A2	4,6-disubstituted 2-deoxystreptamines
2.676	485.3088	484.3036184	-4.3	$C_{26}H_{44}O_8$	[M+H] +	Pseudomonic acid C	Fatty alcohols
4.26	487.2237	486.2186599	-4.58	$C_{18}H_{30}N_8O_8$	[M+H] +	LL-AB 664	Alpha amino acid amides
2.874	489.2023	488.1947366	0.58	$C_{28}H_{28}N_2O_6$	[M+H] +	Sorazolon A2	Carbazoles
3.838	492.2363	491.226765	4.58	$C_{24}H_{33}N_3O_8$	[M+H] +	Bacilosarcin A	2-benzopyrans
3.706	496.2782	495.2733213	-4.83	$C_{28}H_{37}N_3O_5$	[M+H] +	Nerfilin I	Dipeptides
2.135	496.2973	495.2904279	-0.81	$C_{20}H_{41}N_5O_9$	[M+H] +	JI-20B	Aminocyclitol glycosides
2.196	497.2966	496.2896996	-0.75	$C_{24}H_{40}N_4O_7$	[M+H] +	Cytosaminomycin E	Aminoglycosides

Supplementary Table S3. LC/MS metabolite profile of CR1-2 bacterial isolate

R/T. (Min)	Meas.(M/Z)	Calc.(m/z)	Error.ppm	Formulae	Adducts	Compound name	Ontology
2.52	313.1234	312.1143781	3.233	$C_{14}H_{20}N_2O_4S$	[M+H] +	Antibiotic PS 6	Carbapenems
7.914	315.1671	314.1590198	2.550	$C_{13}H_{22}N_4O_5$	[M+H] +	Dapdiamide B zwitterion	Dipeptides
14.103	316.1304	315.121906	3.85	$C_{16}H_{17}N_3O_4$	[M+H] +	Maremycin D1	Alpha amino acids and derivatives
13.907	317.2062	316.199822	-2.83	$C_{15}H_{28}N_2O_5$	[M+H] +	Tenacibactin A	Fatty acid methyl esters
3.283	322.1739	321.1688562	-6.93	$C_{16}H_{23}N_3O_4$	[M+H] +	Streptcytosine L	Hydroxypyrimidines
2.641	322.1739	321.1688562	-6.93	$C_{16}H_{23}N_3O_4$	[M+H] +	Streptcytosine M	Hydroxypyrimidines
8.058	324.1532	323.1481208	-6.78	$C_{15}H_{21}N_3O_5$	[M+H] +	Streptcytosine G	Hydroxypyrimidines
2.869	324.1922	323.1845063	1.29	$C_{16}H_{25}N_3O_4$	[M+H] +	Streptcytosine K	Hydroxypyrimidines
3.759	326.1732	325.1637708	6.60	$C_{15}H_{23}N_3O_5$	[M+H] +	Streptcytosine F	Hydroxypyrimidines
2.523	326.3071	325.2980795	5.35	$C_{20}H_{39}NO_2$	[M+H] +	Semiplenamide C	N-acyl amines
4.199	330.1435	329.1375561	-4.03	$C_{17}H_{19}N_3O_4$	[M+H] +	Mazethramycin	1,4-benzodiazepines
2.439	335.1815	334.1740012	0.66	$C_{14}H_{26}N_2O_7$	[M+H] +	Dihydroactinospectacin	1,4-dioxanes
2.523	337.163	336.1572885	-4.64	$C_{18}H_{24}O_6$	[M+H] +	Dihydrotetrodecamycin	Butenolides
13.984	188.0709	187.0633285	1.57	$C_{11}H_9NO_2$	[M+H] +	Indoleacrylic acid	Indoles
2.876	189.0866	188.0797069	-2.02	$C_7H_{12}N_2O_4$	[M+H] +	tabtoxinine-delta-lactam	Alpha amino acids and derivatives
2.694	346.2237	345.2151377	3.72	$C_{17}H_{31}NO_6$	[M+H] +	Maoxianamide A	Tertiary alcohols
4.25	346.2237	345.2151377	3.72	$C_{17}H_{31}NO_6$	[M+H] +	Maoxianamide B	Tertiary alcohols
2.895	347.1799	346.1740012	-3.97	$C_{15}H_{26}N_2O_7$	[M+H] +	Spenolimycin/Acmimycin	1,4-dioxanes
3.846	195.0766	194.0691422	0.93	$C_9H_{10}N_2O_3$	[M+H] +	Antibiotic XK 90	Hydroxybenzaldehydes
2.196	362.221	361.2113897	6.44	$C_{18}H_{27}N_5O_3$	[M+H] +	Bacithrocin C 1	Amphetamines and derivatives
6.985	197.1289	196.1211778	2.26	$C_{10}H_{16}N_2O_2$	[M+H] +	Amiclenomycin	L-alpha-amino acids
13.984	369.2285	368.2198887	3.62	$C_{20}H_{32}O_6$	[M+H] +	Platenolide B	Macrolides and analogues
2.465	370.2472	369.2416272	-4.60	$C_{22}H_{31}N_3O_2$	[M+H] +	Pendolmycin	Alpha amino acid amides
7.914	373.2085	372.2008846	0.91	$C_{16}H_{28}N_4O_6$	[M+H] +	putrebactin	Macrolactams
2.408	200.0923	199.0844579	2.83	$C_9H_{13}NO_4$	[M+H] +	Anticapsin	L-alpha-amino acids
2.526	378.1281	377.1222999	-3.90	$C_{17}H_{19}N_3O_7$	[M+H] +	Cyanogriside G	Bipyridines and oligopyridines
2.839	384.1566	383.1481208	3.13	$C_{20}H_{21}N_3O_5$	[M+H] +	Pelagiomicin B	Phenazines and derivatives
13.906	384.2641	383.2572773	-1.18	$C_{23}H_{33}N_3O_2$	[M+H] +	Methylpendolmycin	Alpha amino acid amides
13.899	384.2759	383.2671733	3.78	$C_{21}H_{37}NO_5$	[M+H] +	Tumonoic acid F	Proline and derivatives
13.945	386.2278	385.2212857	-1.97	$C_{18}H_{31}N_3O_6$	[M+H] +	Propioxatin B	Dipeptides
9.151	388.1844	387.1794209	-5.92	$C_{20}H_{25}N_3O_5$	[M+H] +	Bioxalomycin alpha1	Piperazinopiperidines
13.899	399.224	398.2165347	0.47	$C_{18}H_{30}N_4O_6$	[M+H] +	cytosamine	Aminoglycosides

3.269	400.1295	399.1212544	2.42	$C_{15}H_{21}N_5O_6S$	[M+H] +	deoxycylindrospermopsin	Pyridopyrimidines
2.889	400.1295	399.1212544	2.42	$C_{15}H_{21}N_5O_6S$	[M+H] +	7-Epicylindrospermopsin	Pyridopyrimidines
13.899	404.1449	403.13795	-0.81	$C_{19}H_{21}N_3O_7$	[M+H] +	Cyanogriside A	Bipyridines and oligopyridines
2.893	406.1839	405.1760668	1.37	$C_{17}H_{23}N_7O_5$	[M+H] +	Cytomycin	Pyrimidones
2.231	407.2202	406.2137578	-2.05	$C_{18}H_{34}N_2O_6S$	[M+H] +	Lincomycin	Proline and derivatives
2.57	408.1966	407.1917169	-5.86	$C_{17}H_{25}N_7O_5$	[M+H] +	Feldamycin	Dipeptides
3.065	411.2003	410.1940679	-2.54	$C_{21}H_{30}O_8$	[M+H] +	Deoxypentalenylglucuron	Terpene glycosides
12.69	414.1274	413.1222999	-5.25	$C_{20}H_{19}N_3O_7$	[M+H] +	Proximicin B	2-heteroaryl carboxamides
9.806	416.1816	415.1743355	-0.03	$C_{21}H_{25}N_3O_6$	[M+H] +	Aclidinomycin A	Isoquinoline quinones
2.523	424.2058	423.2005503	-4.78	$C_{20}H_{29}N_3O_7$	[M+H] +	Amicoumacin A	Beta amino acids and derivatives
6.055	427.2836	426.2770097	-1.60	$C_{27}H_{38}O_4$	[M+H] +	Noscomin	Diterpenoids
2.804	427.2836	426.2770097	-1.60	$C_{27}H_{38}O_4$	[M+H] +	Comnostin E	Sesquiterpenoids
3.903	431.2247	430.2189347	-3.50	$C_{18}H_{26}N_{10}O_3$	[M+H] +	Sinanomycin	Alpha amino acid amides
2.302	437.2141	436.209718	-6.62	$C_{23}H_{32}O_8$	[M+H] +	Luminacin A1	Alkyl-phenylketones
2.329	441.2973	440.2926598	-5.97	$C_{28}H_{40}O_4$	[M+H] +	chaxalactin A	Macrolides and analogues
6.99	444.1988	443.1944023	-6.48	$C_{24}H_{29}NO_7$	[M+H] +	platensin A4	Acylaminobenzoic acid and derivatives
13.878	217.0971	216.0898776	-0.25	$C_{12}H_{12}N_2O_2$	[M+H] +	Mansouramycin A	Isoquinoline quinones
4.243	444.3135	443.3035588	6.00	$C_{27}H_{41}NO_4$	[M+H] +	Piericidin B5	Sesquiterpenoids
2.893	449.226	448.2169286	4.00	$C_{18}H_{32}N_4O_9$	[M+H] +	Fradiamine B	Hydroxy fatty acids
2.585	452.325	451.3198776	-4.76	$C_{28}H_{41}N_3O_2$	[M+H] +	Olivoretin D	Alpha amino acid amides
4.199	454.2305	453.2263711	-6.93	$C_{25}H_{31}N_3O_5$	[M+H] +	moiramide B	Beta amino acids and derivatives
7.911	459.3038	458.2933285	6.95	$C_{30}H_{38}N_2O_2$	[M+H] +	Kenalactam C	Cyclic carboximidic acids
4.757	465.249	464.2410181	1.52	$C_{25}H_{36}O_8$	[M+H] +	Luminacin G2	Sesquiterpenoids
2.862	467.2068	466.2004907	-2.07	$C_{28}H_{26}N_4O_3$	[M+H] +	Staurosporine	Indolocarbazoles
6.055	471.227	470.2224079	-5.69	$C_{17}H_{34}N_4O_{11}$	[M+H] +	Neomycin K	Aminocyclitol glycosides
2.775	476.3059	475.3005987	-4.15	$C_{21}H_{41}N_5O_7$	[M+H] +	Netilmicin	Aminocyclitol glycosides
3.283	481.2854	480.2795289	-2.92	$C_{20}H_{40}N_4O_9$	[M+H] +	Combimicin A2	4,6-disubstituted 2-deoxystreptamines
3.212	482.297	481.290034	-0.64	$C_{23}H_{39}N_5O_6$	[M+H] +	actinomycin x0	Cyclic depsipeptides
2.869	484.269	483.2620879	-0.75	$C_{28}H_{37}NO_6$	[M+H] +	Trienomycin I	1-hydroxy-4-unsubstituted benzenoids
4.193	484.2804	483.2733213	-0.41	$C_{27}H_{37}N_3O_5$	[M+H] +	Acidiphilamide D	Peptides
3.764	494.2993	493.290034	4.03	$C_{24}H_{39}N_5O_6$	[M+H] +	Mullinamide A	Hybrid peptides
2.523	496.2775	495.2733213	-6.24	$C_{28}H_{37}N_3O_5$	[M+H] +	Nerfilin I	Dipeptides
6.055	496.2973	495.2904279	-0.81	$C_{20}H_{41}N_5O_9$	[M+H] +	Antibiotic JI-20B	Aminocyclitol glycosides
12.694	497.2591	496.2533141	-2.99	$C_{23}H_{36}N_4O_8$	[M+H] +	Cytosaminomycin C	Aminoglycosides
2.761	497.2591	496.2533141	-2.99	$C_{23}H_{36}N_4O_8$	[M+H] +	Cytosaminomycin D	Aminoglycosides

2.57	503.2075	502.1991533	2.13	$C_{30}H_{30}O_7$	[M+H] +	Benastatin B	Phenanthrenes and derivatives
2.566	503.3073	502.3002643	-0.48	$C_{23}H_{42}N_4O_8$	[M+H] +	Tenacibactin C	Branched fatty acids
7.393	507.2668	506.2628162	-6.49	$C_{26}H_{38}N_2O_8$	[M+H] +	Macrotermycin C	O-glycosyl compounds
2.889	507.2668	506.2628162	-6.49	$C_{26}H_{38}N_2O_8$	[M+H] +	Macrotermycin D	O-glycosyl compounds
6.985	507.2668	506.2628162	-6.49	$C_{26}H_{38}N_2O_8$	[M+H] +	deformylantimycin a1a	N-acyl-alpha amino acids and derivatives
3.023	511.1821	510.1764012	-3.09	$C_{26}H_{22}N_8O_4$	[M+H] +	guanitrypmycin a2-2	Pyrroloindoles
2.938	515.1758	514.1699784	-2.82	$C_{24}H_{26}N_4O_9$	[M+H] +	Antibiotic TAN 585D	Oligopeptides
5.268	516.2656	515.2631505	-0.20	$C_{27}H_{37}N_3O_7$	[M+H] +	Jomthonic acid D	N-acyl-alpha amino acids
2.336	517.2235	516.2154891	1.42	$C_{24}H_{32}N_6O_5S$	[M+H] +	Aerucyclamide C	Cyclic peptides
2.592	521.2263	520.2209514	-3.70	$C_{29}H_{32}N_2O_7$	[M+H] +	Asukamycin D	Cyclohexenones
2.782	521.2263	520.2209514	-3.70	$C_{29}H_{32}N_2O_7$	[M+H] +	Alisamycin	Cyclohexenones
2.855	529.288	528.2835516	-5.34	$C_{29}H_{40}N_2O_7$	[M+H] +	Hydroxyikarugamycin C	Iridoids and derivatives
2.869	531.2666	530.2628162	-6.57	$C_{28}H_{38}N_2O_8$	[M+H] +	Herbimycin B	Macrolactams
4.25	539.2841	538.2791349	-4.28	$C_{29}H_{38}N_4O_6$	[M+H] +	Dibohemamine F	Pyrrolizines
3.67	542.258	541.2482883	4.49	$C_{21}H_{39}N_3O_{13}$	[M+H] +	Destomycin C	
3.241	549.379	548.3713041	0.76	$C_{32}H_{52}O_7$	[M+H] +	Actinoallolide D	Diterpene lactones
2.666	551.2862	550.2791349	-0.38	$C_{30}H_{38}N_4O_6$	[M+H] +	Dibohemamine E	Pyrrolizines
2.154	551.392	550.3869541	-4.04	$C_{32}H_{54}O_7$	[M+H] +	Spirodienal	Fatty alcohols
2.52	552.1946	551.1869321	0.71	$C_{23}H_{29}N_5O_{11}$	[M+H] +	Herbicidin A	Glycosylamines
7.914	562.3715	561.3665530	-4.14	$C_{32}H_{51}NO_7$	[M+H] +	Nocardiopsin D	Macrolide lactams
13.907	567.3854	566.3818687	-6.60	$C_{32}H_{54}O_8$	[M+H] +	Actinoallolide B	Sesterterpenoids

Supplementary Table S4. LC/MS metabolite profile of CR1-3 bacterial isolate

R/T. (Min)	Meas.(M/Z)	Calc.(m/z)	Error.ppm	Formulae	Adducts	Compound name	Ontology
2.946	313.1234	312.1143	5.58	$C_{14}H_{20}N_2O_4S$	[M+H] +	Antibiotic PS 6	Carbapenems
3.521	315.1671	314.1590	2.55	$C_{13}H_{22}N_4O_5$	[M+H] +	Dapdiamide B zwitterion	Dipeptides
9.581	947.483	946.4800	-4.51	$C_{48}H_{66}N_8O_{12}$	[M+H] +	Lyngbyastatin 7	Cyclic depsipeptides
3.839	950.5303	949.5219	1.18	$C_{42}H_{75}N_7O_{17}$	[M+H] +	sideromycin a	Disaccharides
13.653	251.1387	250.1317	-1.10	$C_{13}H_{18}N_2O_3$	[M+H] +	Bohemamine D	Pyrrolizines
10.371	251.1754	250.1681	0.10	$C_{14}H_{22}N_2O_2$	[M+H] +	Ornilactam A	Tetrahydropyridines
13.251	251.1754	250.1681	0.10	$C_{14}H_{22}N_2O_2$	[M+H] +	Pyrrolizixenamide B	Pyrrolizines
13.798	979.4869	978.4810	-1.40	$C_{47}H_{66}N_{10}O_{13}$	[M+H] +	Cyanopeptolin CP962	Cyclic depsipeptides
4.051	1008.665	1007.651	6.67	$C_{51}H_{89}N_7O_{13}$	[M+H] +	surfactin A	Cyclic depsipeptides
12.921	1022.676	1021.667	1.69	$C_{52}H_{91}N_7O_{13}$	[M+H] +	Norsurfactin	Cyclic depsipeptides
3.276	1022.676	1021.667	1.69	$C_{52}H_{91}N_7O_{13}$	[M+H] +	surfactin B	Cyclic depsipeptides
13.826	1036.694	1035.683	3.59	$C_{53}H_{93}N_7O_{13}$	[M+H] +	surfactin C	Cyclic depsipeptides
2.394	1044.658	1043.650	0.69	$C_{53}H_{93}N_3O_{17}$	[M+H] +	Neocopiamycin A	Macrolides and analogues
2.617	252.2062	251.1997	-3.08	$C_{14}H_{25}N_3O$	[M+H] +	Nocarimidazole B	Aryl alkyl ketones
13.096	1050.709	1049.698	3.54	$C_{54}H_{95}N_7O_{13}$	[M+H] +	Pumilacidin F	Cyclic depsipeptides
8.617	1058.674	1057.666	0.68	$C_{54}H_{95}N_3O_{17}$	[M+H] +	Copiamycin A	Macrolides and analogues
10.038	1063.508	1062.508	-6.84	$C_{44}H_{74}N_{10}O_{20}$	[M+H] +	Aquachelin A	Oligopeptides
13.556	1064.724	1063.714	2.56	$C_{55}H_{97}N_7O_{13}$	[M+H] +	Pumilacidin E	Cyclic depsipeptides
10.98	1067.579	1066.569	2.55	$C_{52}H_{78}N_{10}O_{14}$	[M+H] +	Nostopeptolide A2	Cyclic depsipeptides
2.834	1068.662	1067.650	4.42	$C_{55}H_{93}N_3O_{17}$	[M+H] +	Antibiotic RS 22B	Macrolides and analogues
3.914	1068.662	1067.650	4.42	$C_{55}H_{93}N_3O_{17}$	[M+H] +	Azalomycin F3a	Macrolides and analogues
5.032	1073.677	1072.664	5.33	$C_{52}H_{88}N_{12}O_{12}$	[M+H] +	Pelgipeptin A	Cyclic depsipeptides
4.248	1074.592	1073.592	-6.77	$C_{57}H_{87}NO_{18}$	[M+H] +	Antibiotic 3874H3	Aminoglycosides
3.938	1087.473	1086.467	-1.17	$C_{55}H_{74}O_{22}$	[M+H] +	Landomycin A	Anthraquinones
12.918	1090.701	1089.692	1.58	$C_{55}H_{99}N_3O_{18}$	[M+H] +	Polaramycin A	Macrolides and analogues
10.639	1099.456	1098.448	0.66	$C_{46}H_{66}N_{16}O_{12}S_2$	[M+H] +	Trichamide	Oligopeptides
9.701	1104.716	1103.708	0.66	$C_{56}H_{101}N_3O_{18}$	[M+H] +	Polaramycin B	Macrolides and analogues
13.57	1112.685	1111.674	3.35	$C_{53}H_{93}N_9O_{16}$	[M+H] +	Massetolide E	Cyclic depsipeptides
2.925	1126.702	1125.689	5.08	$C_{54}H_{95}N_9O_{16}$	[M+H] +	Viscosin	Cyclic depsipeptides
3.255	255.1492	254.1419	0.09	$C_{16}H_{18}N_2O$	[M+H] +	Elymoclavine	Clavines and derivatives
8.314	255.1492	254.1419	0.09	$C_{16}H_{18}N_2O$	[M+H] +	Labradorin 2	Indoles

5.748	257.128	256.1211	-1.46	$C_{15}H_{16}N_2O_2$	[M+H] +	albonoursin	Pyrazines
11.657	257.149	256.1423	-2.24	$C_{12}H_{20}N_2O_4$	[M+H] +	pulcherriminic acid	Pyrazinium compounds
2.554	259.1086	258.1004	3.57	$C_{14}H_{14}N_2O_3$	[M+H] +	Antibiotic Tue 2480F1	Harmala alkaloids
3.437	263.103	262.0953	1.61	$C_{13}H_{14}N_2O_4$	[M+H] +	Neothramycin A	Pyrrolo[2,1-c] [1,4] benzodiazepines
3.051	263.103	262.0953	1.61	$C_{13}H_{14}N_2O_4$	[M+H] +	Chicamycin B	Pyrrolo[2,1-c] [1,4] benzodiazepines
3.787	265.1546	264.1473	0.09	$C_{14}H_{20}N_2O_3$	[M+H] +	Bohemamine C	Pyrrolizines
12.167	267.111	266.1055	-6.65	$C_{16}H_{14}N_2O_2$	[M+H] +	Nannozinone B	Pyrrolopyrazines
13.826	267.1353	266.1266	5.53	$C_{13}H_{18}N_2O_4$	[M+H] +	gamma-glutamyltyramine zwitterion	Glutamine and derivatives
5.752	267.1353	266.1266	5.53	$C_{13}H_{18}N_2O_4$	[M+H] +	Jenamidine C	Pyrrolizines
12.791	268.1307	267.1219	5.68	$C_{12}H_{17}N_3O_4$	[M+H] +	Streptcytosine H	Pyrimidones
12.162	269.0896	268.0807	6.04	$C_{10}H_{12}N_4O_5$	[M+H] +	Formycin B	C-glycosyl compounds
2.68	272.1612	271.1532	2.66	$C_{12}H_{21}N_3O_4$	[M+H] +	Vazabitide A	Peptides
2.729	276.1087	275.1018	-1.36	$C_{12}H_{13}N_5O_3$	[M+H] +	5'-Deoxytoyocamycin	Glycosylamines
3.051	279.1559	278.1477	3.31	$C_{11}H_{22}N_2O_6$	[M+H] +	Nitracidomycin A	Gamma amino acids and derivatives
4.517	281.1501	280.1423	1.86	$C_{14}H_{20}N_2O_4$	[M+H] +	Bohemamine E	Pyrrolizines
11.898	283.1044	282.0964	2.56	$C_{11}H_{14}N_4O_5$	[M+H] +	dehydrocoformycin	Glycosylamines
3.051	284.1856	283.1783	0.08	$C_{15}H_{25}NO_4$	[M+H] +	Aerocyanidin	Long-chain fatty acids
4.545	276.0529	275.0463	-2.45	$C_{10}H_{13}NO$	[M+H] +	Streptazone D	Tetrahydropyridines
3.626	290.1713	289.1637	1.12	$C_{12}H_{23}N_3O_5$	[M+H] +	Vazabitide B	Peptides
11.384	291.1708	290.1630	1.80	$C_{16}H_{22}N_2O_3$	[M+H] +	Phthoxazolin A	Fatty amides
11.828	293.148	292.1423	-5.38	$C_{15}H_{20}N_2O_4$	[M+H] +	Antibiotic RK 441	Piperidinediones
3.501	294.2784	293.2718	-2.3	$C_{19}H_{35}NO$	[M+H] +	Lepadiformine	Azaspirodecane derivatives
3.801	295.1194	294.1103	6.18	$C_{15}H_{18}O_6$	[M+H] +	Pentalenolactone O	Terpene lactones
6.344	295.1194	294.1103	6.18	$C_{15}H_{18}O_6$	[M+H] +	Pentalenolactone H	Delta valerolactones
3.135	295.1283	294.1215	-1.61	$C_{14}H_{18}N_2O_5$	[M+H] +	Chicamycin A	1,4-benzodiazepines
12.924	296.1615	295.1532	3.46	$C_{14}H_{21}N_3O_4$	[M+H] +	Streptcytosine J	Hydroxypyrimidines
11.081	296.2213	295.2147	-2.28	$C_{17}H_{29}NO_3$	[M+H] +	Cyclizidine G	Indolizidines
12.792	299.1012	298.0953	-4.6	$C_{16}H_{14}N_2O_4$	[M+H] +	nybomycin	4-quinolinemethanols
11.449	301.1899	300.1837	-3.57	$C_{18}H_{24}N_2O_2$	[M+H] +	Trichostatin RK	Alkyl-phenylketones
11.551	303.1888	302.1841		$C_{14}H_{26}N_2O_5$	[M+H] +	Tenacibactin B	Branched fatty acids
3.037	303.2065	302.1994	-0.58	$C_{18}H_{26}N_2O2$	[M+H] +	Benzastatin I	Aromatic monoterpenoids
3.114	310.1768	309.1688	2.33	$C_{15}H_{23}N_3O4$	[M+H] +	Streptcytosine E	N-arylamides
4.065	313.1222	312.1143	1.99	$C_{14}H_{20}N_2O4S$	[M+H] +	Antibiotic PS 6	Carbapenems
2.189	313.2272	312.2201	0.08	$C_{20}H_{28}N_2O$	[M+H] +	Marinopyrazinone A	Pyrazines
11.985	173.0922	172.0847	-1.29	$C_7H_{12}N_2O3$	[M+H] +	Valanimycin	Alpha amino acids and derivatives

13.84	316.1284	315.1219	-2.46	$C_{16}H_{17}N_3O4$	[M+H] +	Maremycin D1	Alpha amino acids and derivatives
3.374	316.1330	315.1259	-0.06	$C_{21}H_{17}NO2$	[M+H] +	Strepantibin B	Benzene and substituted derivatives
11.341	318.1661	317.1600	-3.69	$C_{14}H_{19}N_7O2$	[M+H] +	Kikumycin B	Proline and derivatives
8.919	321.2049	320.1987	-3.35	$C_{19}H_{28}O_4$	[M+H] +	Agglomerin D	Butenolides
3.184	321.2049	320.1987	-3.35	$C_{19}H_{28}O_4$	[M+H] +	Monacolin J	Delta valerolactones
13.447	325.0846	324.0779	-1.77	$C_{14}H_{16}N_2O_{5S}$	[M+H] +	Asparenomycin C	Carbapenems
13.703	325.1512	324.1433	1.92	$C_{14}H_{20}N_4O_5$	[M+H] +	Resorcinomycin A	N-acyl-alpha amino acids
11.692	327.3023	326.2933	5.27	$C_{19}H_{38}N_2O_2$	[M+H]+	Jietacin B	Ketones
12.785	328.2235	327.2158	1.29	$C_{16}H_{29}N_3O_4$	[M+H]+	Diprotin B	Oligopeptides
3.661	329.1364	328.1310	-5.7	$C_{19}H_{20}O_5$	[M+H]+	Amicolatopsis sp	Hydroxyanthraquinones
13.023	178.0861	177.0789	-0.43	$C_{10}H_{11}NO_2$	[M+H]+	Streptazone A	Epoxypiperidines
3.43	333.1959	332.1888	-0.53	$C_{22}H_{24}N_2O$	[M+H]+	Lavanducyanin	Phenazines and derivatives
5.166	336.2169	335.2096	0.07	$C_{19}H_{29}NO_4$	[M+H]+	Cinnabaramide A	Alpha amino acids and derivatives
4.927	341.1571	340.1481	5.05	$C_{12}H_{24}N_2O_9$	[M+H]+	Fortimicin FU-10	Aminocyclitol glycosides
4.446	344.1596	343.1532	-2.54	$C_{18}H_{21}N_3O_4$	[M+H]+	Porothramycin A	1,4-benzodiazepines
4.58	344.1596	343.1532	-2.54	$C_{18}H_{21}N_3O_4$	[M+H]+	Streptcytosine N	Phenylacetamides
2.932	345.1123	344.1042	2.38	$C_{14}H_{20}N_2O_6S$	[M+H]+	Carpetimycin C	Thienamycins
12.788	354.1892	353.1798	5.99	$C_{13}H_{27}N_3O_8$	[M+H]+	Fortimicin AO	Aminocyclitol glycosides
11.461	354.2256	353.2202	-5.29	$C_{19}H_{31}NO_5$	[M+H]+	Cinnabaramide E	Alpha amino acids and derivatives
2.881	116.0701	115.0633	-4.10	$C_5H_9NO_2$	[M+H]+	Acetamidopropanal	Alpha-hydrogen aldehydes
11.103	362.2205	361.2113	5.31	$C_{18}H_{27}N_5O_3$	[M+H]+	Bacithrocin C 1	Amphetamines and derivatives
13.64	188.1067	187.0997	-1.46	$C_{12}H_{13}NO$	[M+H]+	Streptazone E	Tetrahydropyridines
3.079	370.2484	369.2416	-1.28	$C_{22}H_{31}N_3O_2$	[M+H]+	Pendolmycin	Alpha amino acid amides
13.093	370.3314	369.3242	-0.20	$C_{22}H_{43}NO_3$	[M+H]+	Semiplenamide F	Oxirane carboxylic acids and derivatives
2.778	371.1865	370.1780	3.29	$C_{22}H_{26}O_5$	[M+H] +	Macquarimicin C	Prostaglandins and related compounds
2.946	189.0864	188.0797	-3.04	$C_7H_{12}N_2O_4$	[M+H] +	tabtoxinine-delta-lactam	Alpha amino acids and derivatives
3.521	373.2092	372.2008	3.01	$C_{16}H_{28}N_4O_6$	[M+H] +	putrebactin	Macrolactams
9.581	376.2359	375.2270	4.31	$C_{19}H_{29}N_5O_3$	[M+H] +	Bacithrocin B1	Amphetamines and derivatives
3.839	192.1011	191.0946	-4.03	$C_{11}H_{13}NO_2$	[M+H] +	Streptopyrrolidine	Benzene and substituted derivatives
13.653	384.1975	383.1903	-0.19	$C_{14}H_{29}N_3O_9$	[M+H] +	Sorbistin B	Hexoses
11.377	195.0773	194.0691	4.73	$C_9H_{10}N_2O_3$	[M+H] +	Antibiotic XK 90	Hydroxybenzaldehydes

Supplementary Table S5. LC/MS metabolite profile of CR1-4 bacterial isolate

R/T. (Min)	Meas.(M/Z)	Calc.(m/z)	Error.ppm	Formulae	Adducts	Compound name	Ontology
2.395	247.1448	246.1368278	2.82	$C_{14}H_{18}N_2O_2$	[M+H] +	Bohemamine F	Pyrrolizines
3.657	803.3876	802.38478	-5.55	$C_{36}H_{58}N_4O_{16}$	[M+H] +	Streptovirudin B2	N-acyl-alpha-hexosamines
2.685	803.3876	802.38478	-5.55	$C_{36}H_{58}N_4O_{16}$	[M+H] +	tunicamycin A0	N-acyl-alpha-hexosamines
2.867	805.5471	804.5387633	1.32	$C_{46}H_{76}O_{11}$	[M+H] +	Oligomycin F	Macrolides and analogues
4.255	805.5471	804.5387633	1.32	$C_{46}H_{76}O_{11}$	[M+H] +	44-Homooligomycin A	Macrolides and analogues
4.449	247.1653	246.1579572	0.27	$C_{11}H_{22}N_2O_4$	[M+H] +	Lapstatin	Hybrid peptides
3.84	247.1653	246.1579572	0.27	$C_{11}H_{22}N_2O_4$	[M+H] +	Elaiomycin K	Serine and derivatives
3.706	807.5273	806.5180278	2.47	$C_{45}H_{74}O_{12}$	[M+H] +	21-Hydroxyoligomycin A	Macrolides and analogues
2.135	247.1653	246.1579572	0.27	$C_{11}H_{22}N_2O_4$	[M+H] +	Confluenine C	Hydroxamic acids
6.981	811.3023	810.2907793	5.23	$C_{39}H_{42}N_{10}O_8S$	[M+H] +	Argyrin H	Macrolactams
13.985	815.5159	814.507857	0.94	$C_{43}H_{74}O_{14}$	[M+H] +	Lonomycin C	C-glycosyl compounds
2.395	823.5169	822.5129424	-4.03	$C_{45}H74O_{13}$	[M+H] +	Concanamycin C	Macrolides and analogues
3.657	825.4344	824.4320067	-5.92	$C_{42}H_{60}N_6O_{11}$	[M+H] +	Arylomycin A2	Oligopeptides
2.685	826.3664	825.3571701	2.36	$C_{43}H_{55}NO_{15}$	[M+H] +	Sulfurmycin G	Anthracyclines
2.867	831.3632	830.3558817	0.05	$C_{36}H_{50}N_{10}O_{13}$	[M+H] +	Rhodobactin	Oligopeptides
4.255	843.4929	842.4901903	-5.41	$C_{42}H_{66}N_8O_{10}$	[M+H] +	Marformycin F	Cyclic depsipeptides
4.449	851.5489	850.5442426	-3.08	$C_{47}H_{78}O_{13}$	[M+H] +	Antibiotic A 130C	Sesterterpenoids
3.84	853.3839	852.3806399	-4.71	$C_{44}H_{52}N_8O_{10}$	[M+H] +	pristinamycin IC	Cyclic depsipeptides
3.706	853.3839	852.3806399	-4.71	$C_{44}H_{52}N_8O10$	[M+H] +	Pristinamycin IB	Cyclic depsipeptides
2.135	856.4111	855.4014349	2.79	$C_{41}H_{57}N7O_{13}$	[M+H] +	Arylomycin B1	Oligopeptides
6.981	860.4858	859.4843767	-6.8	$C_{46}H_{65}N_7O_9$	[M+H] +	Xentrivalpeptide A	Cyclic depsipeptides
13.985	867.399	866.3962899	-5.26	$C_{45}H_{54}N_8O_{10}$	[M+H] +	Mikamycin B	Cyclic depsipeptides
7.918	870.5851	869.5738604	4.55	$C_{45}H_{75}N_9O_8$	[M+H] +	Surugamide H	Cyclic peptides
7.432	878.4379	877.4280436	2.94	$C_{37}H_{63}N_7O_{17}$	[M+H] +	Serobactin C	Hybrid peptides
8.059	891.457	890.4446052	5.74	$C_{42}H_{70}N_2O_{16}S$	[M+H] +	Mycinamicin XI	Aminoglycosides
2.191	891.5086	890.5027717	-1.62	$C_{48}H_{74}O_{15}$	[M+H] +	Avermectin B2a	Milbemycins
2.732	917.485	916.4755114	2.41	$C_{48}H_{72}N_2O_{13}S$	[M+H] +	Archazolid D	Macrolides and analogues
2.388	921.4144	920.4127278	-6.08	$C_{41}H_{60}N_8O_{16}$	[M+H] +	Sarpeptin A	Hybrid peptides
2.527	979.4869	978.4810822	-1.49	$C_{47}H_{66}N_{10}O_{13}$	[M+H] +	Cyanopeptolin CP962	Cyclic depsipeptides
7.918	979.4869	978.4810822	-1.49	$C_{47}H_{66}N_{10}O_{13}$	[M+H] +	Micropeptin EI 992	Cyclic depsipeptides
7.432	1022.676	1021.667486	1.21	$C_{52}H_{91}N_7O_{13}$	[M+H] +	Surfactin E	Cyclic depsipeptides
8.059	1022.676	1021.667486	1.21	$C_{52}H_{91}N_7O_{13}$	[M+H] +	surfactin B	Cyclic depsipeptides

4.526	1044.658	1043.650499	0.22	$C_{53}H_{93}N_3O_{17}$	[M+H] +	Neocopiamycin A	Macrolides and analogues
2.225	1050.709	1049.698786	2.8	$C_{54}H_{95}N_7O_{13}$	[M+H] +	Pumilacidin G	Cyclic depsipeptides
2.874	1050.709	1049.698786	2.8	$C_{54}H_{95}N_7O_{13}$	[M+H] +	Pumilacidin F	Cyclic depsipeptides
3.033	1058.674	1057.666149	0.54	$C_{54}H_{95}N_3O_{17}$	[M+H] +	Copiamycin A	Macrolides and analogues
2.564	1064.724	1063.714436	2.15	$C_{55}H_{97}N_7O_{13}$	[M+H] +	Pumilacidin E	Cyclic depsipeptides
9.802	1067.579	1066.569897	1.71	$C_{52}H_{78}N_{10}O_{14}$	[M+H] +	Nostopeptolide A2	Cyclic depsipeptides
12.687	1068.662	1067.650499	3.95	$C_{55}H_{93}N_3O_{17}$	[M+H] +	Antibiotic RS 22B	Macrolides and analogues
2.506	1068.662	1067.650499	3.95	$C_{55}H_{93}N_3O_{17}$	[M+H] +	Azalomycin F3a	Macrolides and analogues
2.527	252.2062	251.1997624	-3.32	$C_{14}H_{25}N_3O$	[M+H] +	Nocarimidazole B	Aryl alkyl ketones
12.737	1073.677	1072.664466	4.9	$C_{52}H_{88}N_{12}O_{12}$	[M+H] +	Pelgipeptin A	Cyclic depsipeptides
13.904	1087.473	1086.467174	-1.33	$C_{55}H_{74}O_{22}$	[M+H] +	Landomycin A	Anthraquinones
13.9	1090.701	1089.692363	1.25	$C_{55}H_{99}N_3O_{18}$	[M+H] +	Polaramycin A	Macrolides and analogues
13.925	1104.716	1103.708013	0.64	$C_{56}H_{101}N_3O_{18}$	[M+H] +	Polaramycin B	Macrolides and analogues
2.858	1112.685	1111.674028	3.32	$C_{53}H_{93}N_9O_{16}$	[M+H] +	Massetolide E	Cyclic depsipeptides
2.17	265.1546	264.1473925	-0.26	$C_{14}H_{20}N_2O_3$	[M+H] +	Bohemamine B	Pyrrolizines
3.79	267.1353	266.1266571	5.12	$C_{13}H_{18}N_2O_4$	[M+H] +	Jenamidine B	Pyrrolizines
2.557	268.1307	267.121906	5.66	$C_{12}H_{17}N_3O_4$	[M+H] +	Streptcytosine H	Pyrimidones
2.648	269.0896	268.0807695	5.78	$C_{10}H_{12}N_4O_5$	[M+H] +	Formycin B	C-glycosyl compounds
4.456	275.1398	274.1317424	2.84	$C_{15}H_{18}N_2O_3$	[M+H] +	Terezine A	Methoxypyrazines
3.075	275.1398	274.1317424	2.84	$C_{15}H_{18}N_2O_3$	[M+H] +	Pinodiketopiperazine A	Alpha amino acids and derivatives
2.984	276.1087	275.1018393	-1.5	$C_{12}H_{13}N_5O_3$	[M+H] +	5'-Deoxytoyocamycin	Glycosylamines
2.528	276.0529	275.0463581	-2.66	$C_{10}H_{13}NO$	[M+H] +	Streptazone D	Tetrahydropyridines
2.527	276.0529	275.0463581	-2.66	$C_{10}H_{13}NO$	[M+H] +	Dihydroabikoviromycin	Epoxypiperidines
12.737	283.1044	282.0964195	2.49	$C_{11}H_{14}N_4O_5$	[M+H] +	dehydrocoformycin	Glycosylamines
13.904	284.1391	283.1320768	-0.89	C16H17N3O2	[M+H] +	Tryptophandehydrobutyrine diketopiperazine	Alpha amino acids and derivatives
13.9	291.1569	290.1477864	6.31	$C_{12}H_{22}N_2O_6$	[M+H] +	Nitracidomycin B	Gamma amino acids and derivatives
13.925	293.148	292.1423071	-5.4	$C_{15}H_{20}N_2O_4$	[M+H] +	Epiderstatin	Piperidinediones
2.858	293.148	292.1423071	-5.4	$C_{15}H_{20}N_2O_4$	[M+H] +	Bripiodionen	Pyrans
2.17	295.1194	294.1103383	6.05	$C_{15}H_{18}O_6$	[M+H] +	Pentalenolactone O	Terpene lactones
3.79	295.1194	294.1103383	6.05	$C_{15}H_{18}O_6$	[M+H] +	Pentalenolactone H	Terpene lactones
2.557	295.1283	294.1215717	-1.86	$C_{14}H_{18}N_2O_5$	[M+H] +	Chicamycin A	1,4-benzodiazepines
2.648	296.161	295.1532062	1.75	$C_{14}H_{21}N_3O_4$	[M+H] +	Streptcytosine J	Hydroxypyrimidines
4.456	299.1012	298.0953569	-4.79	$C_{16}H_{14}N_2O_4$	[M+H] +	nybomycin	4-quinolinemethanols
3.075	301.0938	300.0845175	6.66	$C_{13}H_{16}O_8$	[M+H] +	Pseudolaroside A	Phenolic glycosides
2.984	307.1637	306.1579572	-4.99	$C_{16}H_{22}N_2O_4$	[M+H] +	Phthoxazolin B	Oxazoles

2.528	307.1884	306.1831093	-6.46	$C_{18}H_{26}O_4$	[M+H] +	6-Desmethylmonacolin	Delta valerolactones
4.526	309.1795	308.1736072	-4.47	$C_{16}H_{24}N_2O_4$	[M+H] +	Bestatin	Hybrid peptides
2.225	310.1768	309.1688562	2.15	$C_{15}H_{23}N_3O_4$	[M+H] +	Streptcytosine E	N-arylamides
2.874	173.0922	172.0847922	0.76	$C_7H_{12}N_2O_3$	[M+H] +	Valanimycin	Alpha amino acids and derivatives
3.033	313.1222	312.1143781	1.74	$C_{14}H_{20}N_2O_4S$	[M+H] +	Antibiotic PS 6	Carbapenems
2.564	313.2272	312.2201635	-0.76	$C_{20}H_{28}N_2O$	[M+H] +	Marinopyrazinone A	Pyrazines
9.802	316.1284	315.121906	-2.47	$C_{16}H_{17}N_3O_4$	[M+H] +	Maremycin D1	Alpha amino acids and derivatives

Supplementary Table S6. LC/MS metabolite profile of CR1-5 bacterial isolate

R/T. (Min)	Meas.(M/Z)	Calc.(m/z)	Error.ppm	Formulae	Adducts	Compound name	Ontology
2.519	132.0656	131.0582431	0.61	$C_5H_9NO_3$	[M+H] +	5-Aminolevulinic acid	Delta amino acids and derivatives
14.103	390.1659	389.1586855	-0.16	$C_{19}H_{23}N_3O_6$	[M+H] +	Cyanogriside H	Bipyridines and oligopyridines
8.054	391.2169	390.2114493	-4.67	$C_{16}H_{30}N_4O_7$	[M+H] +	pre-putrebactin	N-acyl amines
2.869	398.2426	397.2365	-2.95	$C_{23}H_{31}N_3O_3$	[M+H] +	Cyclomarazine A	Alpha amino acids and derivatives
3.759	408.3121	407.3035588	3.10	$C_{24}H_{41}NO_4$	[M+H] +	Tricholide A	Macrolide lactams
2.519	411.2	410.1940679	-3.27	$C_{21}H_{30}O_8$	[M+H] +	Deoxypentalenylglucuron	Terpene glycosides
2.435	412.2467	411.2409585	-3.72	$C_{25}H_{33}NO_4$	[M+H] +	Aurachin P	Sesquiterpenoids
13.878	211.1444	210.1368278	1.40	$C_{11}H_{18}N_2O_2$	[M+H] +	L, L-Cyclo(leucylprolyl)	Alpha amino acids and derivatives
2.519	416.1816	415.1743355	-0.03	$C_{21}H_{25}N_3O_6$	[M+H] +	Aclidinomycin A	Isoquinoline quinones
13.983	213.0856	212.0797069	-6.49	$C_9H_{12}N_2O_4$	[M+H] +	surugapyrrole A	Beta amino acids and derivatives
2.876	423.2039	422.1954053	2.88	$C_{23}H_{26}N_4O_4$	[M+H] +	Sevadicin	Oligopeptides
2.694	423.2218	422.2165347	-4.75	$C_{20}H_{30}N_4O_6$	[M+H] +	Glidobactin H	Dipeptides
4.25	215.1378	214.1317424	-5.66	$C_{10}H_{18}N_2O_3$	[M+H] +	Dethiobiotin	Medium-chain fatty acids
13.983	427.2836	426.2770097	-1.60	$C_{27}H_{38}O_4$	[M+H] +	Noscomin	Diterpenoids
2.526	431.2247	430.2189347	-3.50	$C_{18}H_{26}N_{10}O_3$	[M+H] +	Sinanomycin	Alpha amino acid amides
13.906	217.098	216.0898776	3.90	$C_{12}H_{12}N_2O_2$	[M+H] +	Mansouramycin A	Isoquinoline quinones
13.899	432.2408	431.2307878	6.33	$C_{24}H_{33}NO_6$	[M+H] +	Myxopyronin B	Aryl ketones
13.948	432.2823	431.2743839	1.48	$C_{19}H_{37}N_5O_6$	[M+H] +	Istamycin C1	Aminocyclitol glycosides
13.892	433.2232	432.2148034	2.59	$C_{24}H_{32}O_7$	[M+H] +	Chrolactomycin	Furopyrans
13.892	441.2973	440.2926598	-5.97	$C_{28}H_{40}O_4$	[M+H] +	chaxalactin A	Macrolides and analogues
2.231	444.3135	443.3035588	6.00	$C_{27}H_{41}NO_4$	[M+H] +	Piericidin B5	Sesquiterpenoids
9.805	459.3031	458.2933285	5.43	$C_{30}H_{38}N_2O_2$	[M+H] +	Kenalactam C	Cyclic carboximidic acids
12.686	467.2109	466.2004907	6.71	$C_{28}H_{26}N_4O_3$	[M+H] +	Staurosporine	Indolocarbazoles
2.519	468.2838	467.2784067	-4.02	$C_{27}H_{37}N_3O_4$	[M+H] +	26-carboxylyngbyatoxin A	Aromatic monoterpenoids
2.301	481.2854	480.2795289	-2.92	$C_{20}H_{40}N_4O_9$	[M+H] +	Combimicin A2	4,6-disubstituted 2-deoxystreptamines
2.329	484.269	483.2620879	-0.75	$C_{28}H_{37}NO_6$	[M+H] +	Trienomycin I	1-hydroxy-4-unsubstituted benzenoids
6.987	494.2993	493.290034	4.03	$C_{24}H_{39}N_5O_6$	[M+H] +	Syringolin	Dipeptides
13.878	496.2755	495.2692985	-2.17	$C_{23}H_{37}N_5O_7$	[M+H] +	Mullinamide A	Oligopeptides
4.243	496.2954	495.2904279	-4.64	$C_{20}H_{41}N_5O_9$	[M+H] +	Antibiotic JI-20B	Aminocyclitol glycosides
2.89	497.2593	496.2533141	-2.59	$C_{23}H_{36}N_4O_8$	[M+H] +	Cytosaminomycin D	Aminoglycosides
2.673	503.307	502.3002643	-1.07	$C_{23}H_{42}N_4O_8$	[M+H] +	Tenacibactin C	Branched fatty acids
7.907	504.2432	503.2379984	-4.11	$C_{24}H_{33}N_5O_7$	[M+H] +	Norplicacetin	Aminoglycosides

13.878	504.3395	503.3359215	3.28	$C_{28}H_{45}N_3O_5$	[M+H] +	majusculamide A	Dipeptides
4.187	508.2744	507.265459	-1.57	$C_{27}H_{41}NO_6S$	[M+H] +	Epothilone B	Epothilones and analogues
2.869	510.2512	509.2447236	-1.57	$C_{26}H_{39}NO_7S$	[M+H] +	Epothilone A9	Epothilones and analogues
3.759	510.2512	509.2447236	6.24	$C_{26}H_{39}NO_7S$	[M+H] +	Epothilone M	Macrolides and analogues
12.693	516.2664	515.2631505	1.42	$C_{27}H_{37}N_3O_7$	[M+H] +	Jomthonic acid D	N-acyl-alpha amino acids
2.561	517.2235	516.2154891	-5.34	$C_{24}H_{32}N_6O_5S$	[M+H] +	Aerucyclamide C	Cyclic peptides
2.883	529.288	528.2835516	5.28	$C_{29}H_{40}N_2O_7$	[M+H] +	Hydroxyikarugamycin	Iridoids and derivatives
3.135	530.3868	529.3767237	-4.20	$C_{32}H_{51}NO_5$	[M+H] +	Myxotyroside A	O-glycosyl compounds
3.016	533.3774	532.3723666	3.72	$C_{27}H_{52}N_2O_8$	[M+H] +	Deinococcucin A	N-acyl-alpha-hexosamines
5.264	535.2571	534.2478348	-4.29	$C_{29}H_{34}N_4O_6$	[M+H] +	DKxanthene-534	Asparagine and derivatives
2.336	539.2841	538.2791349	4.49	$C_{29}H_{38}N_4O_6$	[M+H] +	Dibohemamine F	Pyrrolizines
7.956	542.258	541.2482883	4.49	$C_{21}H_{39}N_3O_{13}$	[M+H] +	Destomycin C	2-deoxystreptamine aminoglycosides
2.855	542.258	541.2482883	4.49	$C_{21}H_{39}N_3O_{13}$	[M+H] +	KA-5685	Aminocyclitol glycosides
2.869	549.379	548.371304	0.76	$C_{32}H_{52}O_7$	[M+H] +	Actinoallolide D	Diterpene lactones
4.25	551.392	550.3869541	-4.04	C32H54O7	[M+H] +	Spirodienal	Fatty alcohols
2.666	567.3854	566.3818687	-6.60	$C_{32}H_{54}O_8$	[M+H] +	Actinoallolide B	Sesterterpenoids
2.294	579.2938	578.2839455	4.45	$C_{29}H_{42}N_2O_{10}$	[M+H] +	Geldanamycin F	Long-chain fatty acids
2.54	586.314	585.3050153	2.91	$C_{31}H_{43}N_3O_8$	[M+H] +	tanespimycin	Macrolactams
7.914	593.4032	592.3975188	-2.69	$C_{34}H_{56}O_8$	[M+H] +	Irumanolide II	Diterpene lactones
4.39	597.3034	596.2958476	0.46	$C_{30}H_{40}N_6O_7$	[M+H] +	Spumigin F	N-acyl-alpha amino acids and derivatives
13.92	603.4079	602.4003127	0.52	$C_{28}H_{54}N_6O_8$	[M+H] +	Tenacibactin D	N-acyl amines
2.105	606.4556	605.45162	-5.43	$C_{33}H_{59}N_5O_5$	[M+H] +	Dragonamide B	Valine and derivatives
3.486	607.4227	606.4131688	3.71	$C_{35}H_{58}O_8$	[M+H] +	21-deoxybafilomycin A1	Macrolides and analogues
3.605	244.1085	243.1007767	1.83	$C_{13}H_{13}N_3O_2$	[M+H] +	caerulomycin J	Bipyridines and oligopyridines
2.519	621.425	620.4149001	4.54	$C_{33}H_{56}N_4O_7$	[M+H] +	Rakicidin B	Cyclic depsipeptides
14.103	621.425	620.4149001	4.54	$C_{33}H_{56}N_4O_7$	[M+H] +	Rakicidin I	Cyclic depsipeptides
8.054	623.2813	622.269752	6.85	$C_{25}H_{42}N_4O_{14}$	[M+H] +	Allosamidin	N-acyl-alpha-hexosamines
2.869	624.3157	623.309432	-1.61	$C_{35}H_{45}NO_9$	[M+H] +	Proansamycin B	Macrolactams
3.759	624.3157	623.309432	-16.1	$C_{35}H_{45}NO_9$	[M+H] +	Chaxamycin B	Macrolactams
2.519	635.3038	634.2962415	0.44	$C_{29}H_{42}N_6O_{10}$	[M+H] +	Oxamycetin	Aminoglycosides
2.435	646.3223	645.312226	4.33	$C_{30}H_{43}N_7O_9$	[M+H] +	Actinoramide F	Leucine and derivatives
13.878	654.2907	653.2836112	-0.29	$C_{35}H_{43}NO_{11}$	[M+H] +	Rifamycin W-hemiacetal	Naphthoquinones
2.519	663.2962	662.2852829	5.49	$C_{37}H_{38}N_6O_6$	[M+H] +	Xenematide	Hybrid peptides
13.983	665.2984	664.2915501	-0.64	$C_{26}H_{44}N_6O_{14}$	[M+H] +	Muracein A	Dipeptides
2.876	666.3092	665.3047406	-4.23	$C_{33}H_{47}NO_{13}$	[M+H] +	Natamycin	Aminoglycosides

2.694	670.3383	669.33067	0.53	$C_{25}H_{43}N_{13}O_9$	[M+H] +	Tuberactinomycin O	Cyclic peptides
4.25	673.4292	672.4210481	1.30	$C_{35}H_{56}N_6O_7$	[M+H] +	Nocardiamide B	Oligopeptides
13.983	675.3433	674.3349356	1.61	$C_{34}H_{50}N_4O_8S$	[M+H] +	Microginin 674	Peptides
2.526	693.4825	692.472415	4.05	$C_{37}H_{64}N_4O_8$	[M+H] +	Veraguamide I	Cyclic depsipeptides
13.906	701.3267	700.320725	-1.86	$C_{36}H_{48}N_2O_{12}$	[M+H] +	Alldimycin B	Anthracyclines
13.899	701.3719	700.3670065	-3.40	$C_{35}H_{56}O_{14}$	[M+H] +	Chalcomycin	Macrolides and analogues
13.948	701.3992	700.3908106	1.59	$C_{34}H_{52}N_8O_8$	[M+H] +	Desotamide D	Oligopeptides
13.892	708.3342	707.3240365	4.08	$C_{38}H_{49}N_3O_8S$	[M+H] +	Thiazinotrienomycin D	N-acyl-alpha amino acids and derivatives
2.883	709.5223	708.5176339	-3.68	$C_{41}H_{72}O_9$	[M+H] +	Ionomycin	Diterpene glycosides
13.892	731.3857	730.3775712	1.17	$C_{36}H_{58}O_{15}$	[M+H] +	Aldgamycin I	Macrolides and analogues
2.231	743.4934	742.4813569	6.41	$C_{32}H_{62}N_{12}O_8$	[M+H] +	Argimicin A	Peptides
2.89	770.34	769.3309553	2.30	$C_{40}H_{51}NO_{14}$	[M+H] +	Streptovaricin C	Macrolactams
9.805	777.3824	776.3803651	-6.74	$C_{33}H_{56}N_6O_{15}$	[M+H] +	Nocardamin glucuronide	Macrolactams
12.686	789.3788	788.3691317	3.03	$C_{35}H_{56}N_4O_{16}$	[M+H] +	Streptovirudin A2	N-acyl-alpha-hexosamines
2.519	805.5516	804.5387633	6.90	$C_{46}H_{76}O_{11}$	[M+H] +	Oligomycin F	Macrolides and analogues
2.301	805.5516	804.5387633	6.90	$C_{46}H_{76}O_{11}$	[M+H] +	44-Homooligomycin A	Macrolides and analogues
2.329	261.1237	260.1160924	1.27	$C_{14}H_{16}N_2O_3$	[M+H] +	Maculosin	Alpha amino acids and derivatives
6.987	870.4233	869.4170849	-1.22	$C_{42}H_{59}N_7O_{13}$	[M+H] +	Arylomycin B3	Oligopeptides
13.878	1050.709	1049.698786	2.80	$C_{54}H_{95}N_7O_{13}$	[M+H] +	Pumilacidin G	Cyclic depsipeptides
4.243	1050.709	1049.698786	2.80	$C_{54}H_{95}N_7O_{13}$	[M+H] +	surfactin D	Cyclic depsipeptides
2.89	1058.674	1057.666149	0.54	$C_{54}H_{95}N_3O_{17}$	[M+H] +	Copiamycin A	Macrolides and analogues
2.673	1064.724	1063.714436	2.15	$C_{55}H_{97}N_7O_{13}$	[M+H] +	Pumilacidin E	Cyclic depsipeptides
